# Research Progress on Defect Behavior, Microstructure, and Service Performance of Additively Manufactured Austenitic Stainless Steels

**DOI:** 10.3390/ma19204371

**Published:** 2026-10-17

**Authors:** Kaiyu Liu, Hongbiao Dong, Lijuan Zhang

**Affiliations:** 1 School of Materials Science and Engineering, Shandong University of Technology, Zibo 255049, China; 25609010064@stumail.sdut.edu.cn; 2 School of Metallurgy and Materials, University of Birmingham, Birmingham B15 2TT, UK

**Keywords:** additive manufacturing, austenitic stainless steel, defects, microstructure regulation, service performance

## Abstract

Additive manufacturing provides a promising route for the precision fabrication and property tailoring of austenitic stainless steels because of its high design freedom, rapid customization capability, and near-net-shape manufacturing advantages. However, rapid melting–solidification and non-equilibrium thermal cycling generate strongly textured columnar grains, dislocation-cell structures, retained δ-ferrite, and heterogeneous residual stresses. These features are often accompanied by pores, lack-of-fusion defects, cracks, and environment-sensitive degradation, which restrict reliable application in demanding engineering fields. This review critically summarizes recent progress in defect formation, microstructural regulation, and service performance of additively manufactured austenitic stainless steels, following a unified process–thermal history–defect/microstructure–property framework. Particular attention is given to the effects of solidification mode, crystallographic texture, grain-boundary characteristics, and post-processing on multiscale microstructures and process–defect–microstructure–performance relationships. Finally, key technical bottlenecks, scientific knowledge gaps, and future development directions are identified to guide further research and engineering applications.

## 1. Introduction

### 1.1. Additive Manufacturing Technology

**Figure 1:**
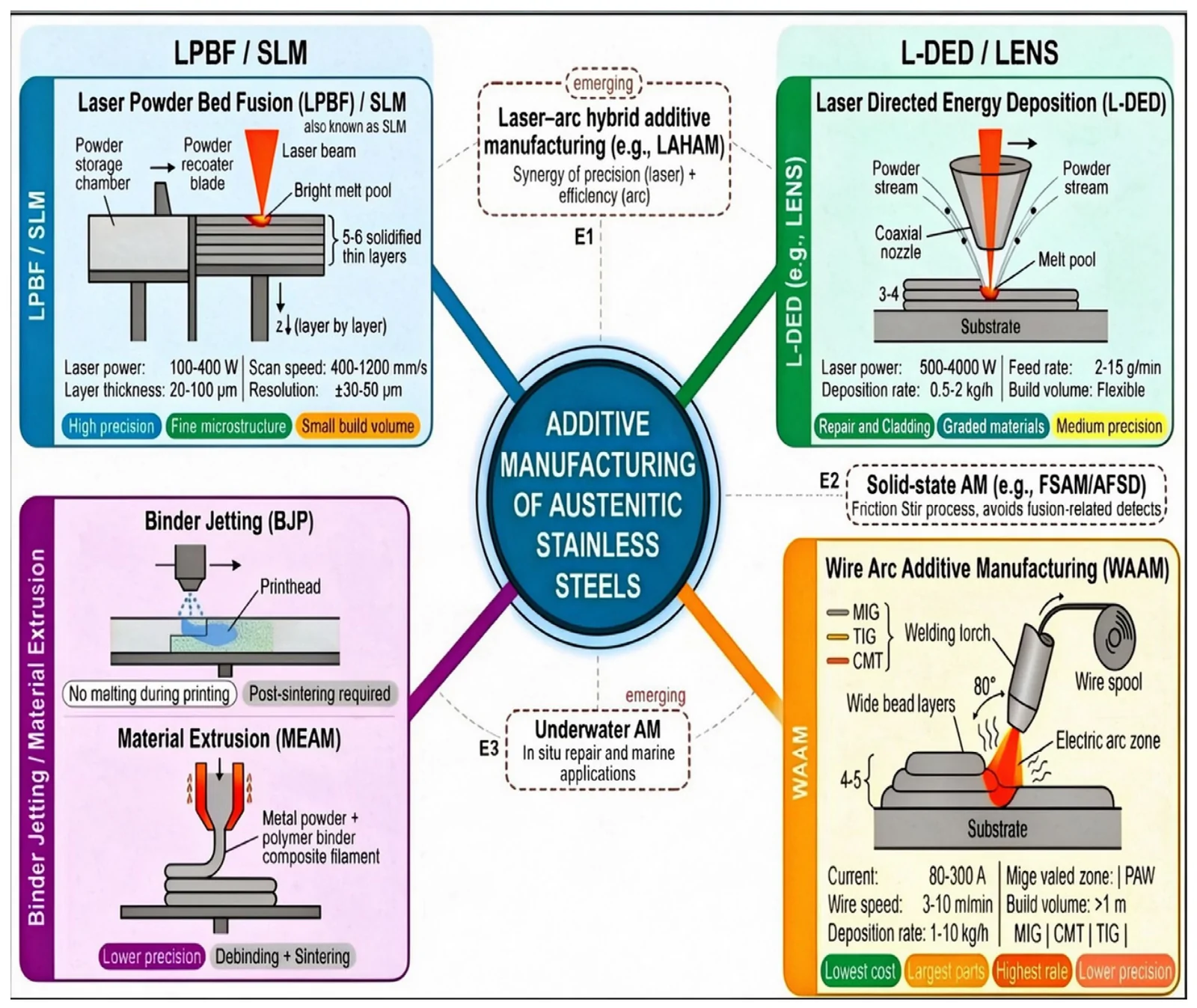
Mainstream technologies of additive manufacturing. Note: AI was used only for text-based conceptual suggestions; the figure was fully created by the authors.

Additive manufacturing, commonly known as 3D printing, is an advanced technology that fabricates three-dimensional objects by sequentially adding material layers based on digital models [1,2]. The fundamental principle, “discretization–accumulation,” involves slicing a 3D model into a series of 2D layers. Energy sources like lasers or electron beams scan each layer, selectively solidifying or sintering powder, melting wire materials, and ultimately forming the object through this layered accumulation process. Unlike traditional subtractive manufacturing, 3D printing bypasses the constraints of molds and complex processing, allowing for the direct and rapid transition from design to product [3,4,5]. Figure 1 illustrates the current mainstream technologies, which include Laser Additive Manufacturing (LAM) [6,7,8], Wire Arc Additive Manufacturing (WAAM) [9,10,11], Binder Jet Printing (BJP) [12,13] and Material Extrusion Additive Manufacturing (MEAM) [14,15,16]. The materials employed in these processes range from polymers and metals to ceramics and even biological materials.

Additive manufacturing, a transformative production technology, offers unprecedented freedom in product design. It efficiently and precisely fabricates complex geometric structures and internal cavities that traditional processes struggle to achieve, enabling topological optimization and lightweighting of structures. This capability has promoted significant advances in aerospace, biomedicine, automotive manufacturing, and design-oriented applications [1,4]. In aerospace, metal additive manufacturing optimizes key components like engine nozzles and satellite brackets [17,18,19]. In the medical field, it allows for the precise preparation of personalized implants, surgical guides, and tissue engineering scaffolds [20,21]. In automotive manufacturing, it provides highly personalized components, such as interior parts and door handles [22,23]. Additionally, additive manufacturing supports defense, space exploration, and bioengineering [24,25,26]. Despite its advantages, the technology faces challenges, including limited build efficiency for large-scale components in some AM processes, particularly powder-bed-based techniques, the need to expand material systems, and the need to improve the consistency of surface quality and mechanical properties. Looking ahead, advancements in multi-material printing, hybrid manufacturing (combining additive and subtractive technologies) and intelligent, sustainable materials are expected to accelerate the transition of additive manufacturing from rapid prototyping to the direct production of functional components. This evolution is anticipated to further integrate into intelligent manufacturing systems, reshaping traditional production modes.

### 1.2. Introduction to Austenitic Stainless Steel

**Figure 2:**
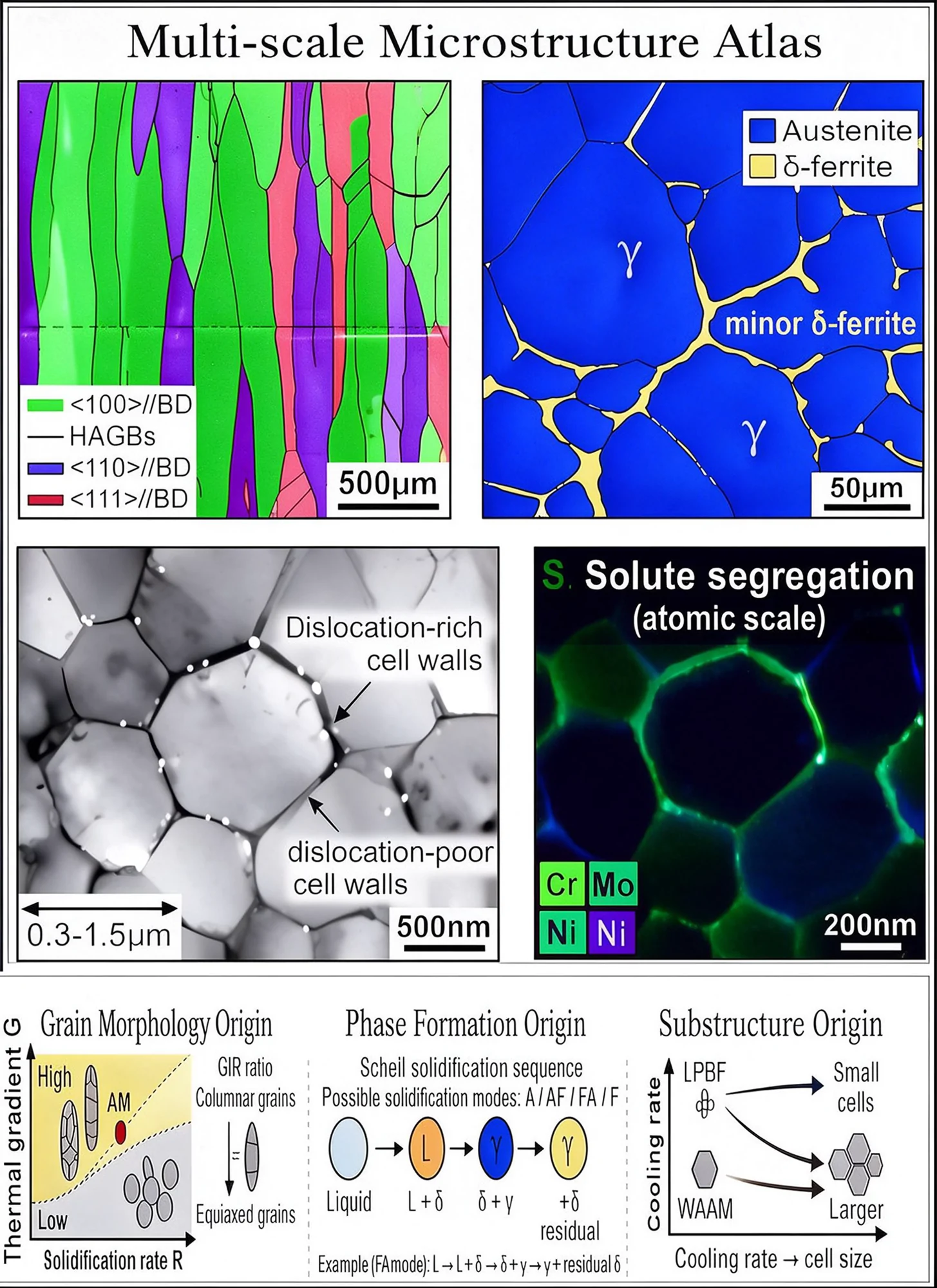
Grades, microstructural characteristics, and thermal-cycle features of additively manufactured austenitic stainless steels. The schematic was initially drafted with the assistance of AI and subsequently redrawn and finalized by the authors.

Austenitic stainless steel is the most commonly used series in modern industry, primarily due to its face-centered cubic (FCC) crystal structure, which is stabilized by elements like nickel, manganese, or nitrogen. Typically, this steel contains 16–20% chromium and 8–12% nickel. Common grades include the general-purpose 304 (18% chromium, 8% nickel) and 316, which offers enhanced pitting corrosion resistance through the addition of 2–3% molybdenum. Low-carbon versions, such as 304L, 316L, and 308L, are developed to improve weldability. Special grades like 321/347 and nickel-saving 201 incorporate stabilizing elements such as titanium or niobium to prevent chromium carbide formation [27,28]. Table 1 details the specific grades and chemical compositions. Due to its excellent corrosion resistance and formability, austenitic stainless steel is a key material in metal additive manufacturing (AM). The relationships among process, microstructure, and properties have been systematically evaluated, as shown in Figure 2 and Table 2.

With advancements in active “additive manufacturing-adapted” alloy design, research is increasingly focusing on systems that significantly enhance performance through innovative alloying. High-nitrogen austenitic stainless steels, for instance, benefit from nitrogen alloying, which synergistically boosts interstitial solid solution strengthening and corrosion resistance. Similarly, super austenitic alloy steels, designed with multi-element alloying including high molybdenum, high chromium, and nickel, exhibit both excellent corrosion resistance and high-temperature strength. These areas have emerged as key directions in the study of high-performance stainless steels for additive manufacturing.

#### 1.2.1. Research Status of Additive Manufacturing of High-Nitrogen Austenitic Stainless Steel

High-nitrogen austenitic stainless steel is an innovative material that enhances the austenite phase structure by incorporating nitrogen, typically exceeding 0.4%, as a substitute for nickel. This steel boasts high strength, toughness, and corrosion resistance, while also being cost-effective, and is extensively used in protective armor and offshore platform structures [54,55,56]. Research on the additive manufacturing of high-nitrogen steel has primarily explored the interplay between manufacturing processes, microstructure evolution, and mechanical property regulation. Techniques such as laser powder bed fusion, arc additive manufacturing, and composite manufacturing have systematically elucidated the effects of nitrogen solid solution behavior, second-phase precipitation mechanisms, and defect control strategies on material properties [57,58]. In microstructure regulation, Tao et al. [59] optimized laser energy density, scanning strategy, and nitrogen protection atmosphere during the laser powder bed fusion process, achieving a nitrogen content of 1.1 wt%. This optimization allowed precise control of the γ-austenite/δ-ferrite phase balance, which was regulated by varying the α-ferrite fraction from 0.5% to 44.9%, thereby promoting solid-solution strengthening and increasing the dislocation density. This provides a viable process window for strengthening and toughening high-nitrogen steel. Additionally, Qian et al. [57] used cold metal transfer arc additive manufacturing to produce high-nitrogen steel, demonstrating a notable strain rate strengthening effect. The dynamic compressive strength ranged from 1303 to 1672 MPa as the strain rate increased, confirming the positive impact of high-nitrogen solid solution on mechanical properties under dynamic loading. However, the as-built microstructure often deviates from equilibrium and requires optimization through post-heat treatment. Zhang et al. [60] conducted isothermal aging research on WAAM high-nitrogen chromium manganese steel, revealing precipitation kinetics. At 900 °C, the microstructure largely transformed into a brittle σ phase with Cr_2_N and secondary austenite precipitation. Raising the temperature above 1000 °C inhibited σ phase precipitation, leading to the dissolution of interdendritic ferrite through element diffusion, stabilizing the microstructure. This clarified the heat treatment window’s regulation mechanism on phase transformation paths and mechanical properties. Besides heat treatment, droplet transfer behavior significantly influences microstructure stability during formation. Zhang et al. [61] achieved stable transfer in CMT welding using a low peak current of 133–234 A and a short peak duration of 3–5 ms, avoiding spatter accumulation in the weld toe area. They found manganese evaporation to be highly sensitive to peak current, peaking at 1.83 wt%. When manganese loss exceeded 1%, the nitrogen loss rate surpassed 20%, shifting the solidification mode from full austenite to an austenite–ferrite dual-phase microstructure, with coarsened oxide inclusions and expanded dendrite spacing. This revealed the coupling between alloying element burning loss and microstructure evolution. To mitigate these defects, Qian et al. [62] introduced an additive–subtractive composite manufacturing process, mechanically removing nitrogen pore-enriched surface areas. This refined columnar austenite grains, disrupted strong texture orientation, elongated granular ferrite radially, and dispersed second-phase particles, reducing defect volume by about 54% and maximum pore diameter by 46.6%. This offers an effective path for enhancing high-nitrogen steel additive manufacturing quality. These studies have laid a crucial foundation for advancing the technological maturity and application of high-nitrogen steel additive manufacturing through process parameter optimization, precise microstructure regulation, and comprehensive performance improvement.

#### 1.2.2. Research Status of Additive Manufacturing of Super Austenitic Alloy Stainless Steel

Super austenitic stainless steels are a class of highly alloyed austenitic stainless steels, typically containing relatively high levels of elements such as chromium (>20%), nickel (>18%), and molybdenum (>3.5%). Their key characteristic lies in achieving significantly superior corrosion resistance compared to traditional austenitic stainless steels like 304 and 316 by substantially increasing the alloy content (especially nickel, chromium, molybdenum, and nitrogen). They exhibit excellent resistance to pitting corrosion and stress corrosion cracking, particularly in harsh acidic, chloride, and seawater environments [63,64,65]. Super austenitic stainless steels have important applications in fields such as chemical engineering, marine, oil and gas, and pollution control. However, their high alloy content results in an extremely stable austenite phase, making it difficult to refine the grains through traditional deformation-induced martensitic transformation and subsequent annealing. Moreover, they are prone to the precipitation of harmful intermetallic compounds such as the σ phase during hot working or medium-temperature heat treatment, which affects toughness and corrosion resistance [65,66]. Previous studies have mainly focused on obtaining ultrafine-grained/nanocrystalline structures through severe plastic deformation (such as high-pressure torsion and equal-channel angular pressing) combined with controlled annealing processes to simultaneously enhance strength and plasticity [67,68,69,70], and to explore new ways to achieve the synergistic optimization of mechanical properties while maintaining excellent corrosion resistance. Currently, additive manufacturing technology, through its unique rapid melting and solidification and complex thermal cycling history, enables materials to form multi-level, non-equilibrium microstructures that differ from those produced by traditional casting and forging processes [71,72,73,74]. For example, Vishnukumar et al. [75] directly formed 904L super austenitic stainless steel components using arc additive manufacturing technology and found that the materials prepared by this process not only have excellent pitting corrosion resistance and stable passivation film formation characteristics but also have a very low degree of intergranular sensitization, fully verifying the feasibility of additive manufacturing technology in the near-net shaping of corrosion-resistant alloys. Pan et al. [76] proposed a technical route of synergistic strengthening by laser cladding and electropolishing. After preparing a N08926 super austenitic stainless steel cladding layer on a Q231 carbon steel substrate, they found that laser cladding effectively inhibits the formation of harmful precipitates and forms fine subgrain structures due to its extremely high solidification rate, while electropolishing further smoothens the surface through selective dissolution, enriches the proportion of high-valence oxides such as Fe^3+^, Cr^3+^ and Mo^6+^, and improves the stability of the passivation film. The synergistic effect of the two makes the composite-treated samples have the lowest corrosion current density (5.38 nA·cm^−2^), the widest passivation range (0.8665 V), and the most stable passivation film. However, single-phase austenitic structures often face a performance trade-off of decreased plasticity while achieving high strength, making it difficult to simultaneously achieve high strength and high ductility. To break through this bottleneck, Fang et al. [77] mixed AL-6XN super austenitic stainless steel powder with 2507 super duplex stainless steel powder and micron TiC particles. Through in situ reactions during the laser powder bed fusion process, TiC_x_N_y_ nanoparticles were generated, which in turn induced a high proportion of Σ3 twin boundaries and nano-twins, and successfully constructed a heterogeneous bimodal grain structure with coexisting coarse austenite and fine austenite/ferrite. This achieved a synergistic improvement in strength and plasticity while maintaining excellent corrosion resistance. The above studies systematically demonstrate multiple technical routes for improving the performance of super austenitic stainless steels and jointly reveal the core role of multi-scale, multi-mechanism synergistic strengthening, toughening, and corrosion resistance in breaking through traditional performance trade-offs.

### 1.3. Summary and Framework

Additive manufacturing offers an efficient route for producing austenitic stainless steel components for marine engineering, energy, nuclear power, and chemical engineering applications owing to its advantages in near-net-shape forming, integrated fabrication of complex structures, and local property customization. Compared with conventional forging, rolling, and casting, AM involves rapid melting–solidification, strong non-equilibrium thermal histories, and repeated thermal cycling. These factors alter solidification pathways, crystallographic orientation, interfacial characteristics, and residual-stress distributions, leading to unique multiscale microstructures and manufacturing defects. Austenitic stainless steels are traditionally valued for their excellent ductility, toughness, and corrosion resistance. Under AM conditions, however, they do not simply retain the microstructure–property relationships of conventionally processed counterparts. Instead, surface and internal defects interact with heterogeneous microstructures to affect local electrochemical uniformity, crack-initiation sensitivity, and mechanical response. These interactions become more complex in chloride-containing environments, molten salts, high-temperature cyclic loading, or hydrogen-containing conditions. Despite significant progress in process optimization, microstructure regulation, and service-performance evaluation, existing studies often report inconsistent conclusions because of variations in manufacturing processes, alloy compositions, heat-input conditions, post-processing routes, and testing environments. A unified framework linking process thermal history, defect evolution, microstructural heterogeneity, and service performance is still lacking.

Therefore, this review establishes a process–thermal history–defect/microstructure–property framework for discussing the core scientific issues and technical challenges in the additive manufacturing of austenitic stainless steels. First, it categorizes common defects in AM austenitic stainless steels and analyzes their formation mechanisms and property effects, with emphasis on hydrogen-embrittlement sensitivity, surface and internal defects, and defect-related microstructural abnormalities. Second, it examines microstructural formation and evolution under the rapid solidification and cyclic thermal histories inherent to AM, focusing on the solidification mode, crystallographic orientation, grain-boundary characteristics, and post-processing regulation of multiscale microstructures. Third, it discusses service performance in terms of corrosion resistance, wear resistance, and fatigue/fracture behavior, and clarifies the relationships among process characteristics, defects, microstructure, environmental factors, post-processing, and service response.

Accordingly, this review follows the logical progression from defect formation to microstructural evolution and service-performance response. The primary aim is to elucidate the intrinsic relationships among process thermal history, defect evolution, microstructural heterogeneity, and service performance, while identifying key technological directions and development trends for overcoming future performance bottlenecks.

### 1.4. Literature Search and Selection

This review is based on a systematic literature search conducted across three databases—Web of Science, Scopus, and ScienceDirect—covering publications from 2000 to 2026. The search employed core terms including additive manufacturing/3D printing, austenitic stainless steel, defects, microstructure, grain-boundary engineering, post-processing, corrosion, wear, fatigue, and hydrogen embrittlement. The initial search yielded over 480 publications; after excluding studies focused primarily on welding and cladding, approximately 400 remained for analysis.

This review does not directly pool or aggregate data obtained under different conditions. Instead, it takes austenitic stainless steel as the unified research subject and is structured along the core logical thread of defect formation–microstructural evolution–service performance, treating alloy grades, additive manufacturing (AM) processes, density, surface condition, and heat treatment as conditional variables within this framework. Specifically, Section 2 discusses defects such as porosity, lack of fusion, and cracks, comparing their formation mechanisms in the context of the thermal histories and melt-pool characteristics of LPBF, WAAM, and DED. Section 3 addresses microstructural evolution in terms of solidification mode, crystallographic orientation, grain-boundary characteristics, and post-processing regulation, accounting for variations in alloy composition and processing conditions. Section 4 analyzes corrosion, wear, and fatigue performance, treating microstructure, surface state, and heat treatment as conditional variables. In cross-condition synthesis, only trend-level conclusions supported by consistent mechanistic explanations are extracted, and specimen state and testing conditions are specified in the text and table captions. For datasets with overly divergent conditions that cannot be integrated into a single mechanistic framework, they are discussed separately rather than averaged quantitatively. Through this approach, heterogeneous studies can be rationally integrated within a unified framework, and the application adaptability and interoperability of AM austenitic stainless steels across different service scenarios can be further discussed, thereby broadening their application scope in additive manufacturing.

During the preparation of this manuscript, the authors used DeepSeek-V3.0, an AI-assisted tool, to generate initial conceptual sketches for several schematic figures. These AI-generated drafts were used solely as references for layout and conceptual design. All final figures were manually redrawn, refined, and finalized by the authors to ensure scientific accuracy. No AI-generated visual content was directly incorporated into the final manuscript. The authors have thoroughly reviewed all AI-assisted output and take full responsibility for the content of this publication.

## 2. Defects in Additively Manufactured Austenitic Stainless Steels

Additive manufacturing enables the fabrication of geometrically complex austenitic stainless steel components. However, the rapid melting and solidification, together with repeated non-equilibrium thermal cycles, often introduce both structural and microstructural defects. These defects can degrade the mechanical properties, dimensional accuracy, corrosion resistance, and long-term reliability of AM-fabricated components. In aggressive service environments, such defects may further promote localized corrosion, corrosion fatigue, stress corrosion cracking, and hydrogen-assisted degradation. Within the process–thermal history–defect/microstructure–property framework, this chapter critically examines how AM thermal history and melt-pool dynamics lead to surface, internal, and microstructural defects as well as hydrogen embrittlement susceptibility in austenitic stainless steels, and how these features affect service performance.

### 2.1. Surface and Internal Structural Defects

**Figure 3:**
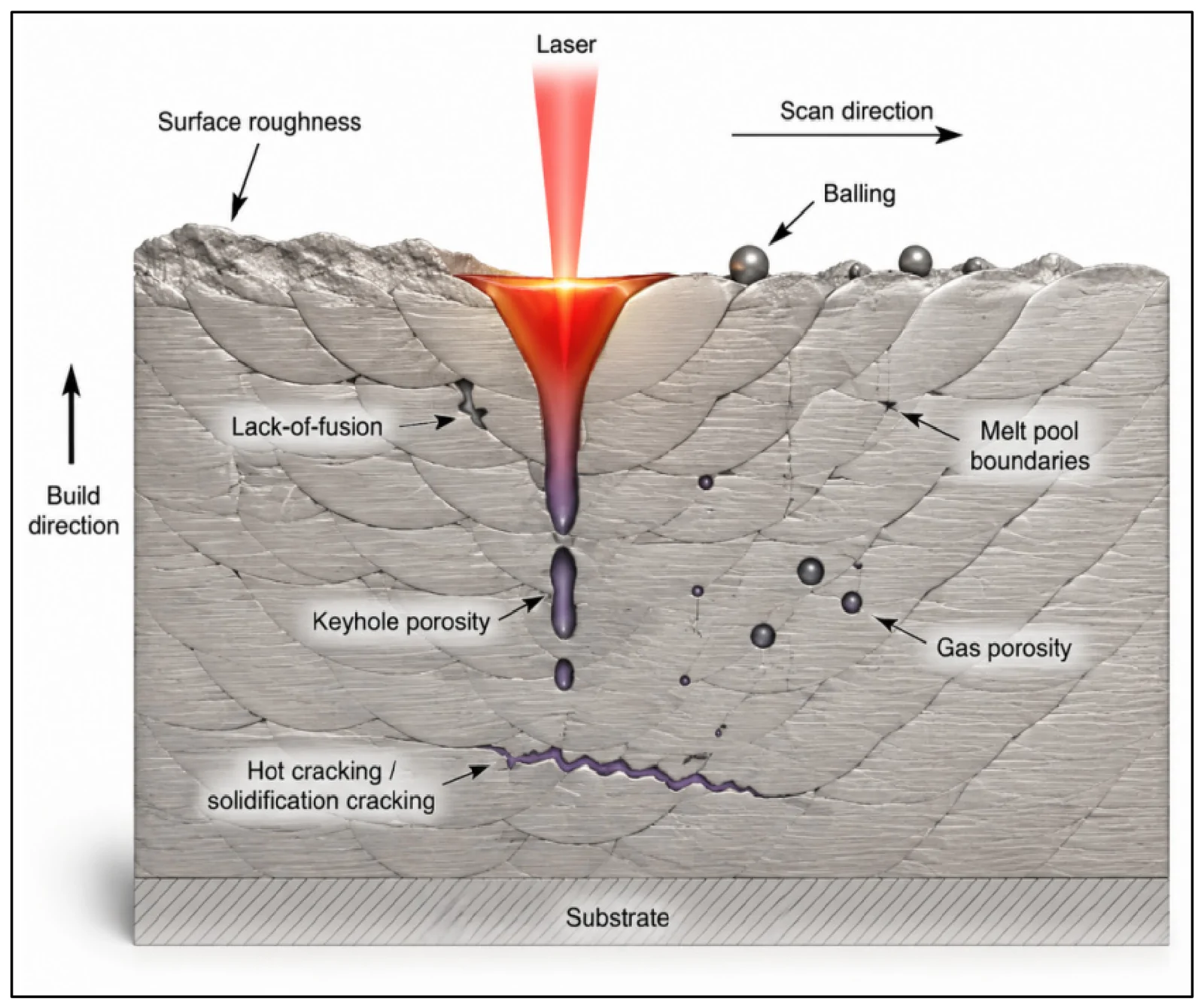
Schematic illustration of typical surface and internal defects in additively manufactured austenitic stainless steels. Note: AI was used only for text-based conceptual suggestions; the figure was fully created by the authors.

Surface and internal defects are among the most critical defect categories affecting the quality and reliability of AM-fabricated austenitic stainless steel components. As shown in Figure 3, surface defects alter the local geometry and induce stress concentrations, thereby compromising dimensional accuracy and surface integrity. Meanwhile, internal defects, such as pores, lack-of-fusion defects, and solidification cracks and thermal-stress-induced cracks, disrupt material continuity and act as preferential sites for crack initiation and propagation. Thus, analyzing the typical characteristics and formation mechanisms at both the macroscopic surface and internal structural levels is essential.

Typical surface defects in AM-fabricated austenitic stainless steels include balling, staircase effects, adhered particles, spatter-induced defects, and surface undulations caused by melt-pool instability [78,79]. Balling is generally associated with pore wettability, insufficient energy input, excessive scanning speed, and melt-pool instability. Under these conditions, the molten track breaks up into discrete droplets driven by surface tension and Plateau–Rayleigh instability. These droplets subsequently solidify into spherical beads that adhere to the build surface or remain trapped between adjacent layers [80]. The staircase effect originates from the discrete layer-by-layer fabrication strategy and leads to stepped deviations on inclined or curved surfaces [81]. Molten pool undulation results from intense Marangoni convection, evaporation recoil pressure, and uneven heat accumulation in overlapping melt tracks, causing periodic peaks and valleys on the melt pool surface. These surface defects directly impair the dimensional accuracy and surface finish of parts. They act as stress concentration points, accelerate oxidation, alter the composition and morphology of the surface oxide film, and significantly reduce the fatigue life of components [82]. In corrosive environments, these defects serve as retention areas for corrosive media, such as chloride ions, accelerating the initiation and propagation of local corrosion, including pitting and crevice corrosion. Additionally, rough surfaces often contain subsurface micropores and microcracks, further compromising the material’s compactness and integrity.

Internal defects in materials primarily include pores and cracks. Pore-related defects can be classified into gas pores, keyhole-induced pores, and lack-of-fusion defects according to their formation mechanisms and morphologies [83,84,85,86]. Gas pores typically originate from entrapped shielding gas, gas trapped within powder particles, adsorbed moisture or surface contaminants, and the vaporization of low-boiling-point alloying elements. These gas bubbles may be retained in the solidified structure because of the short lifetime and rapid solidification of the melt pool. Such pores are generally small and nearly spherical, reflecting their gas-bubble origin. When the energy input is insufficient, owing to high scanning speeds, low energy density, large hatch spacing, or excessive layer thickness, the melt pool becomes too shallow or discontinuous to fully fuse adjacent tracks or layers. This condition leads to irregular, flat, and sharp-edged lack-of-fusion defects. Conversely, excessive energy input, resulting from high laser power or low scanning speed, can induce keyhole-mode melting and melt-pool instability. The collapse of the keyhole cavity may entrap vapor or shielding gas, leading to the formation of keyhole-induced pores. This instability leads to fluctuations or collapse of the keyhole wall, trapping steam at the solidification front and forming irregular, smooth, deep holes at the bottom of the melt pool, a phenomenon common in laser additive manufacturing [80,87,88,89,90]. The type, shape, and distribution of pores significantly affect material properties. Irregular, sharp lack-of-fusion pores notably reduce static strength, ductility, fatigue strength, and corrosion resistance due to high stress concentration [82,91,92]. Interestingly, under certain conditions—such as low pore volume fraction and uniformly distributed near-spherical pores—materials can exhibit a lower steady-state creep rate than fully dense materials by altering dislocation movement. However, if the pore volume fraction exceeds a critical value or the pore shape is irregular, stress concentration negates these benefits, drastically reducing creep life [90,93].

Crack defects in AM-fabricated austenitic stainless steels mainly include solidification cracks, liquation cracks, and cold cracks associated with residual stresses, hydrogen-assisted embrittlement, or strain-induced martensitic transformation [72]. Solidification cracks usually form in the terminal stage of solidification, particularly within the mushy zone where interdendritic liquid films persist. During rapid solidification, solute elements such as Nb and Mo, as well as low-melting-point impurities such as S and P, may segregate to interdendritic regions and grain boundaries, depending on the alloy composition. Such segregation promotes the formation of low-melting-point liquid films along interdendritic regions or grain boundaries, which possess limited mechanical cohesion during the final stage of solidification. When tensile stresses generated by solidification shrinkage and steep thermal gradients exceed the cohesive strength of these liquid films, cracks initiate and propagate along grain boundaries [94,95]. In contrast, cold cracks are generally associated with high residual stresses, hydrogen-assisted cracking, and, in metastable austenitic stainless steels, local strain-induced martensitic transformation [96]. Longitudinal cracks extending across multiple layers along the build direction act as severe stress concentrators and provide preferential pathways for corrosive media. Crack defects severely impair ductility, fatigue life, and fracture toughness. They also accelerate stress corrosion cracking and pose a significant threat, thereby compromising the structural integrity and long-term service reliability of load-bearing components.

Surface and internal defects remain critical challenges for additively manufactured austenitic stainless steels. These defects not only reduce the relative density and dimensional accuracy but also provide preferential sites for localized corrosion, wear damage, and fatigue crack initiation. Therefore, defect mitigation should not be limited to detection and statistical characterization. It is essential to improve melt-pool stability and optimize scanning strategies, build paths, and processing parameters to enhance component quality at the source.

### 2.2. Microstructural Defects

**Figure 4:**
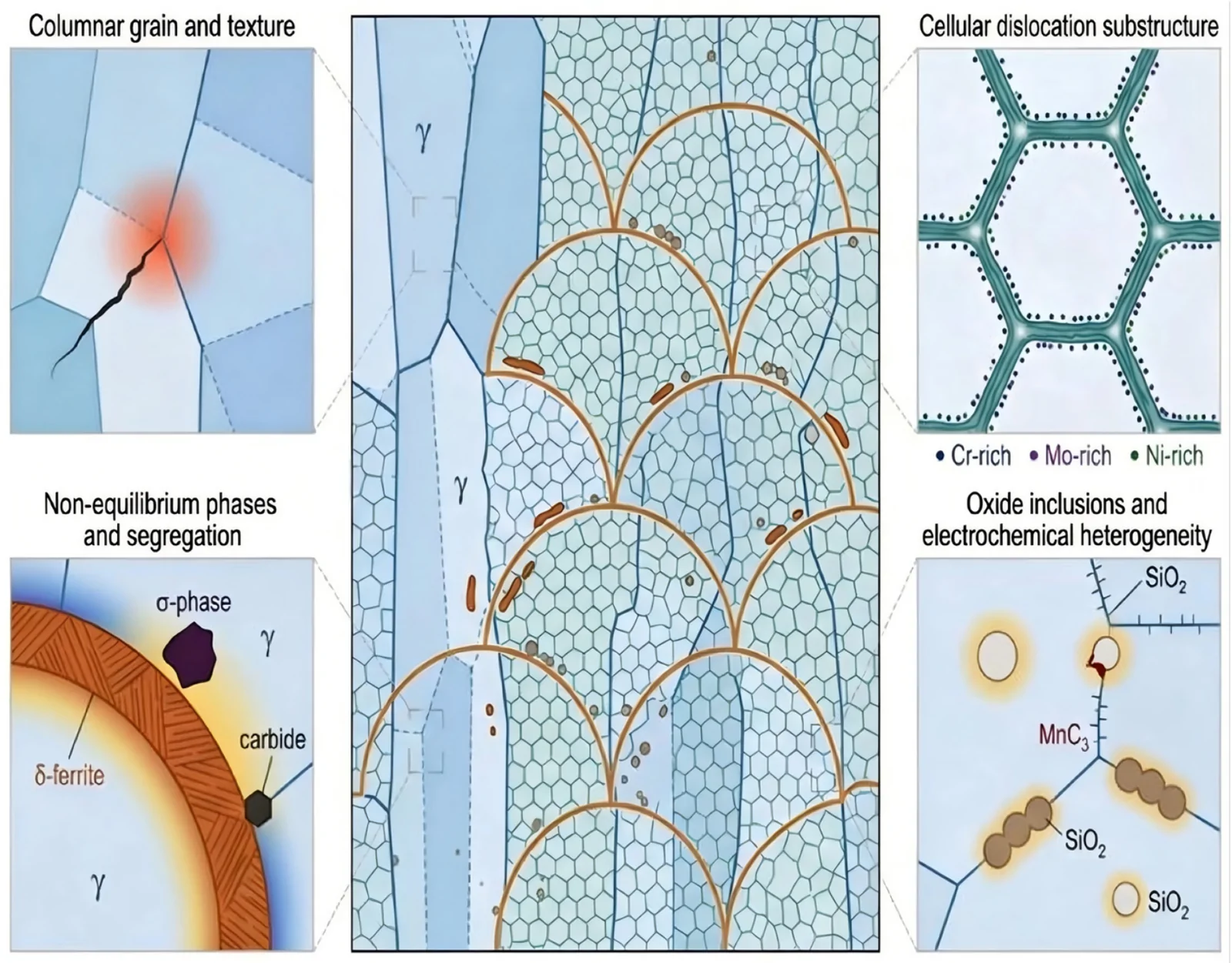
Schematic illustration of typical microstructural defects in additively manufactured austenitic stainless steels, including abnormal grain structures, cellular substructures, elemental segregation, non-equilibrium phases, and oxide inclusions. Note: AI was used only for text-based conceptual suggestions; the figure was fully created by the authors.

During the additive manufacturing of austenitic stainless steels, rapid melting and solidification, steep thermal gradients, and repeated thermal cycles lead to microstructural evolution that differs markedly from that observed in conventionally processed materials. As shown in Figure 4, the resulting microstructural defects mainly include abnormal grain structures, non-equilibrium phases, elemental segregation, and oxide inclusions. However, it should be emphasized that these features are not necessarily defects in all cases. Cellular dislocation structures, in particular, are a characteristic feature of rapidly solidified AM microstructures rather than a defect, and can contribute to strength and strain hardening. Residual δ-ferrite, non-equilibrium phases, elemental segregation, and oxide inclusions exhibit a strong dependence on volume fraction, morphology, size, distribution, and service environment, and may be beneficial or detrimental accordingly. For example, a moderate amount of δ-ferrite can reduce solidification cracking susceptibility, whereas excessive or continuous δ-ferrite networks degrade corrosion resistance and cryogenic toughness. Dispersed oxide inclusions can impede dislocation motion and contribute to dispersion strengthening, while clustered, coarse, or poorly bonded inclusions may act as preferential sites for localized corrosion and fatigue crack initiation. Similarly, elemental segregation and non-equilibrium phases can either enhance strength through solute drag and precipitation or promote localized corrosion and embrittlement depending on their scale and distribution. Their formation mechanisms, evolution, and effects on properties are analyzed separately in the following sections.

1. Abnormal grain structures and substructures. The melt-pool boundary often serves as a heterogeneous nucleation site and promotes epitaxial grain growth along the direction of the maximum thermal gradient, which is typically close to the build direction. This process often produces coarse columnar grains spanning multiple deposited layers and a pronounced crystallographic texture [83,97,98,99,100]. Such coarse columnar grains and strong textures can lead to anisotropic mechanical behavior and crystallographic-orientation-dependent corrosion responses. They may also reduce transverse toughness and resistance to crack propagation, particularly when cracks propagate along columnar grain boundaries. High-energy grain boundaries can act as preferential sites for crack initiation and propagation under thermal or mechanical loading [38,91]. Cellular substructures with characteristic sizes of approximately 0.2–2 μm commonly develop within grains. These cellular substructures are characterized by dense dislocation networks, with cell walls enriched in dislocations and segregated solute elements [71,83,101,102,103].
2. Non-equilibrium phases and elemental segregation. The rapid cooling and repeated reheating inherent to additive manufacturing can promote the retention of non-equilibrium phases and the precipitation of secondary phases, such as residual δ-ferrite, σ phase, and carbides, Residual δ-ferrite may influence phase stability and corrosion behavior, whereas σ phase and carbides can deteriorate ductility and corrosion resistance by promoting local Cr depletion and brittle fracture [104,105,106,107]. Elemental microsegregation occurs when solute elements, such as Cr, Mo, Ni, and Mn, are rejected by the advancing solidification front during rapid solidification. Such segregation is particularly pronounced at cell walls, cellular subgrain boundaries, and melt-pool boundaries [71,108,109]. These non-equilibrium phases and elemental segregation can compromise the long-term reliability and service life of additively manufactured austenitic stainless steel components. They may promote localized corrosion, intergranular corrosion, phase instability, and brittle cracking.
3. Oxide inclusions and impurity enrichment. Oxide inclusions and impurity enrichment primarily originate from native oxide layers, such as SiO_2_, Cr_2_O_3_, and MnCr_2_O_4_, present on the surfaces of powder particles or feedstock wires [110,111,112,113]. They can also form through reactions between molten metal and residual oxygen in the shielding atmosphere, even under nominally inert gas protection. Owing to the extremely high temperature gradients and rapid solidification rates, these oxides have insufficient time to float out of the melt pool before solidification. Consequently, they are retained in the solidified microstructure as nanoscale inclusions, often smaller than 100 nm, with spherical, clustered, or chain-like morphologies. These inclusions are preferentially distributed at interfacial regions, such as melt-pool boundaries, cellular subgrain boundaries, and grain boundaries. Simultaneously, microsegregation of elements such as Si, Mn, and Al may occur [111,112,113]. Although dispersed oxide inclusions may contribute to dispersion strengthening by impeding dislocation motion and pinning grain boundaries, they can compromise the electrochemical uniformity of the matrix, particularly when accompanied by solute-enriched regions. This can promote the formation of local micro-galvanic cells and weak sites in the passive film, potentially increasing the susceptibility to pitting corrosion, intergranular corrosion, and stress corrosion cracking [71,114].

These defects do not exist independently; instead, their formation and evolution are jointly governed by solidification behavior, melt-pool dynamics, thermal cycling, residual stress, and alloy composition. Overall, defect formation in AM-fabricated austenitic stainless steels is governed by the coupling of feedstock quality, energy input, melt-pool dynamics, solidification behavior, and thermal cycling. Surface defects mainly affect dimensional accuracy and fatigue performance, whereas internal pores and cracks directly reduce structural integrity. Microstructural defects, including segregation, non-equilibrium phases, and oxide inclusions, further modify local mechanical and electrochemical responses. Therefore, defect mitigation requires integrated control of powder or wire quality, shielding atmosphere, processing parameters, scanning strategy, and post-processing treatments.

### 2.3. Hydrogen Embrittlement

Austenitic stainless steels are generally considered resistant to hydrogen embrittlement (HE) because their FCC austenitic matrix combines high hydrogen solubility with low hydrogen diffusivity. This resistance is further supported by a low Md30 temperature and relatively high stacking fault energy, which suppress deformation-induced martensitic transformation, and by a hydrogen diffusion coefficient approximately three orders of magnitude lower than that in BCC ferritic steels, which limits rapid hydrogen migration toward crack tips [115,116,117]. However, AM introduces a microstructure that differs fundamentally from wrought austenitic stainless steels. Rapid solidification and repeated thermal cycling generate high-density dislocation cells, residual δ-ferrite, elemental segregation, nanoscale oxides, and heterogeneous residual stresses. These features modify hydrogen trapping, transport, and local accumulation, thereby altering HE susceptibility. A rigorous understanding of HE in AM austenitic stainless steels therefore requires separate consideration of hydrogen trapping, hydrogen transport, and hydrogen-assisted fracture.

**Figure 5:**
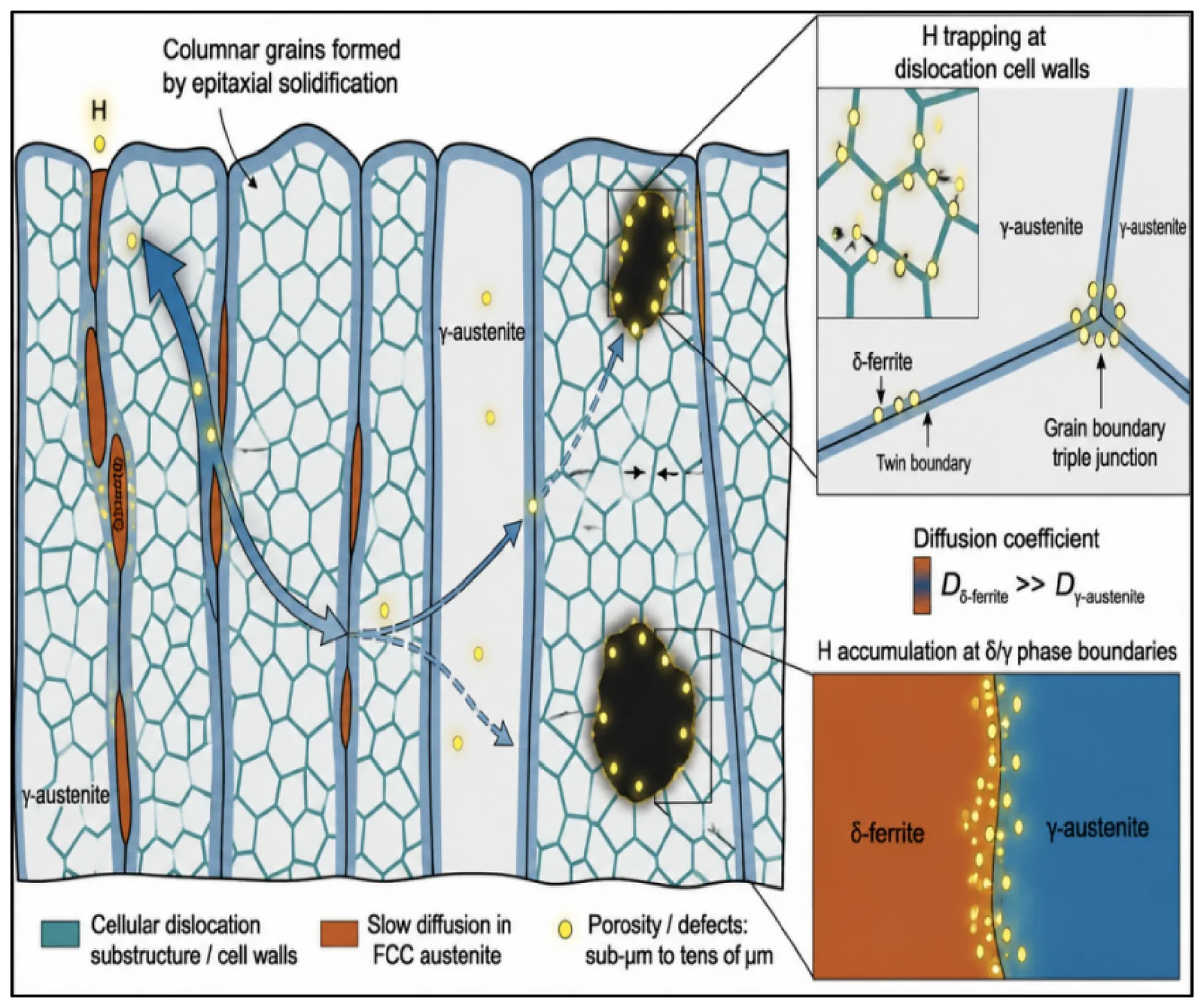
Schematic illustration of hydrogen diffusion and trapping mechanisms in additively manufactured austenitic stainless steels. Note: AI was used only for text-based conceptual suggestions; the figure was fully created by the authors.

Hydrogen trapping in AM austenitic stainless steels is commonly classified into reversible and irreversible traps according to binding energy and room-temperature stability. Reversible traps, such as dislocations, elastic stress fields, and certain grain or phase boundaries, bind hydrogen weakly and can release it back to the lattice at room temperature. Irreversible traps, such as precipitate/matrix interfaces, vacancy clusters, and strongly binding boundaries, retain hydrogen even at room temperature and effectively remove it from the diffusible population. In AM microstructures, high-density dislocation cell walls, δ-ferrite/γ interfaces, melt-pool boundaries, and nanoscale oxide inclusions are all potential trapping sites, but whether each acts as a reversible or irreversible trap depends on its local binding energy and occupancy, which must be determined experimentally; for example, by thermal desorption spectroscopy (TDS). This distinction is critical because reversible and irreversible traps influence HE in opposite ways: reversible traps can either retard hydrogen diffusion or, when locally saturated, release hydrogen to promote cracking, whereas irreversible traps primarily reduce the amount of diffusible hydrogen available for crack-tip accumulation. For instance, dislocation-cell structures in AM 316L have been experimentally shown to act as hydrogen traps that retard hydrogen diffusion toward crack tips and partially restrict dislocation motion, thereby reducing hydrogen-induced damage under certain conditions [71,118,119,120]. In contrast, elemental segregation and phase constitution changes induced by AM can locally alter the M d30temperature and increase HE susceptibility [121,122]. The distinction between reversible and irreversible traps and their influence on hydrogen transport are schematically summarized in Figure 5.

Hydrogen transport in AM microstructures is governed by the spatial distribution and saturation state of these traps. Trapping does not directly determine fracture; it modulates hydrogen transport and local concentration, which in turn controls the driving force for hydrogen-assisted damage. Because traps temporarily or permanently remove hydrogen from the diffusible population, they reduce the apparent hydrogen diffusivity at the macroscopic scale. However, this reduction is not uniformly beneficial. Traps that are saturable and reversible can become local hydrogen sources once their occupancy exceeds a critical level, releasing hydrogen into the surrounding matrix and creating localized hydrogen enrichment. Irreversible traps, by contrast, permanently remove hydrogen from circulation and thus reduce the amount available for crack-tip accumulation. In AM microstructures, the spatial distribution of traps is highly heterogeneous: dislocation cell walls, δ/γ interfaces, and oxide inclusions are concentrated in specific regions, so hydrogen transport is not uniform but follows preferential pathways and accumulates at specific microstructural sites. For example, residual δ-ferrite formed during AM is often distributed along grain boundaries, and because hydrogen diffusivity is higher in ferritic phases than in austenite, such δ-ferrite can provide fast diffusion pathways that promote local hydrogen accumulation and crack initiation [123,124,125]. This effect may partially compromise the intrinsic HE resistance of the FCC austenitic matrix. Some studies suggest that δ-ferrite may improve HE resistance by suppressing deformation twinning and altering local deformation behavior [126]; however, when the δ-ferrite volume fraction is below approximately 10%, its influence on the overall HE response is often considered limited, although local effects may still exist. Transport effects therefore control how much hydrogen reaches a critical site, but they do not by themselves determine whether fracture occurs.

Taken together, the role of dislocation-cell structures and hydrogen traps is condition-dependent: at low occupancy, traps reduce the apparent hydrogen diffusivity and delay hydrogen accumulation at crack tips, thereby mitigating hydrogen-induced damage; once locally saturated, however, they can act as hydrogen sources that promote local enrichment and cracking. The net effect depends on trap density, binding energy, occupancy, and service environment.

**Figure 6:**
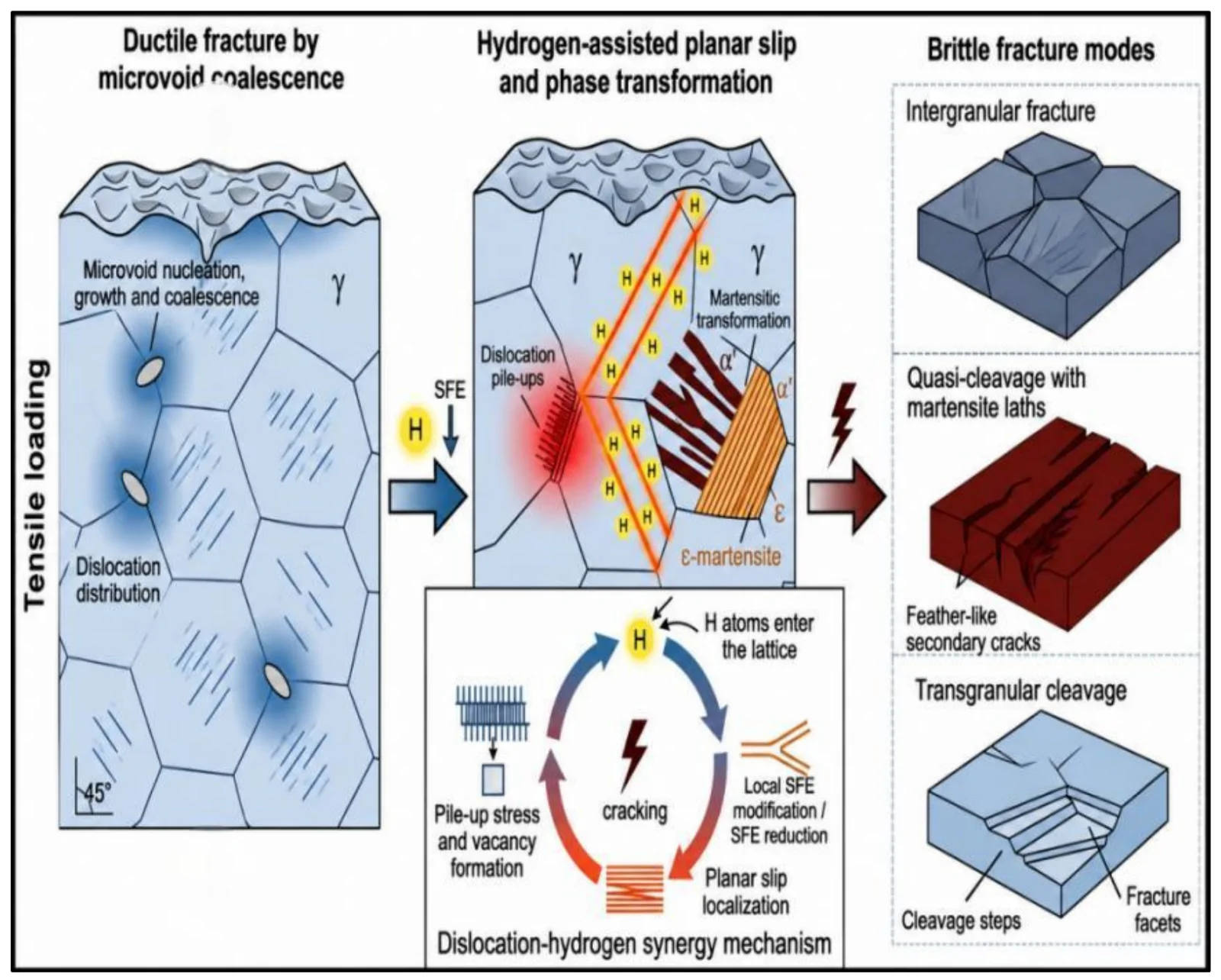
Schematic illustration of the evolution of hydrogen embrittlement fracture modes and hydrogen–dislocation interaction mechanisms. The schematic was initially drafted with the assistance of DeepSeek and subsequently redrawn and finalized by the authors. Note: AI was used only for text-based conceptual suggestions; the figure was fully created by the authors.

Hydrogen-assisted fracture in austenitic stainless steels is generally interpreted using three mechanisms: hydrogen-enhanced localized plasticity (HELP) [127,128], hydrogen-enhanced decohesion (HEDE) [128,129], and hydrogen-enhanced strain-induced vacancy formation (HESIV) [121,130]. These mechanisms describe how hydrogen locally weakens interatomic cohesion, enhances localized plasticity, or promotes vacancy formation, thereby initiating cracks. They are not mutually exclusive and may operate synergistically during deformation and fracture. Microstructural features such as high-density dislocations, grain boundaries, twin boundaries, cellular substructure boundaries, and precipitates influence these mechanisms by acting as hydrogen trapping sites and by modifying local stress and strain fields [117,127]. Once the local hydrogen concentration exceeds a critical level, hydrogen may weaken interatomic cohesion, enhance localized plasticity, or promote vacancy formation, thereby initiating cracks. In austenitic stainless steels, hydrogen can also reduce stacking fault energy, promoting the formation of ε-martensite or α′-martensite during deformation, which markedly reduces ductility and changes the fracture mode from ductile microvoid coalescence to quasi-cleavage along martensite, grain boundaries, or twin boundaries [122,131,132,133]. Because hydrogen diffuses more rapidly in martensitic phases than in austenite, deformation-induced martensite can provide preferential diffusion pathways and promote hydrogen accumulation at martensite/austenite interfaces, facilitating brittle crack propagation [115]. In severe cases, fully brittle transgranular or intergranular fracture may occur [134,135]. The evolution of hydrogen embrittlement fracture modes and the underlying hydrogen-dislocation interaction mechanisms are schematically illustrated in Figure 6.

A clear distinction must be made between what has been experimentally demonstrated for AM microstructures and what has been inferred from conventional austenitic stainless steels. Experimentally, dislocation-cell structures in AM 316L have been shown to act as hydrogen traps and to retard hydrogen diffusion [71,118,119,120]. Residual δ-ferrite in AM microstructures has been observed to provide fast diffusion pathways and to promote local hydrogen accumulation [123,124,125]. These are AM-specific experimental findings. In contrast, the dominance of HELP, HEDE, or HESIV in AM microstructures is largely inferred from conventional austenitic stainless steels, because direct experimental evidence for these mechanisms in AM-specific microstructures—with their unique dislocation cells, residual δ-ferrite, and oxide inclusions—remains limited. This mismatch between conventional and AM-specific HE behavior is closely related to the non-equilibrium solidification, solute segregation, and thermal cycling histories discussed in Section 3.1. Similarly, the role of deformation-induced martensite in promoting brittle fracture has been well established in conventional metastable austenitic stainless steels, but whether the same mechanism dominates in AM austenitic stainless steels depends on the local phase stability, which is itself modified by AM-induced segregation and residual stress. Therefore, AM-specific experimental verification is still needed before conventional HE mechanisms can be assumed to dominate in AM materials.

Characterizing hydrogen traps and transport in AM microstructures requires complementary techniques. Bulk hydrogen concentration methods include TDS, carrier gas hot extraction, and amperometry [136,137,138]. TDS is the primary technique used to quantify the reversible/irreversible trap distinction outlined above, because desorption peaks at different temperatures can distinguish traps with different binding energies and quantify their respective occupancies. Local hydrogen distribution can be characterized using glow discharge optical emission spectroscopy (GDOES), secondary ion mass spectrometry (SIMS), and atom probe tomography (APT) [139,140,141]. These techniques, combined with microstructural characterization by EBSD, TEM, and APT, are essential for determining which specific AM microstructural features act as reversible or irreversible traps and for quantifying their influence on hydrogen transport and fracture.

Overall, defects and environment-assisted degradation in AM austenitic stainless steels should not be regarded as isolated phenomena; rather, they are closely linked to solidification pathways, thermal cycling histories, and local microstructural evolution. Many issues commonly described as defects are fundamentally associated with grain morphology, phase constitution, dislocation substructures, and crystallographic texture. Therefore, the following chapter discusses the formation, evolution, and regulation of microstructures in AM austenitic stainless steels.

## 3. Microstructure Regulation and Analysis of Additively Manufactured Austenitic Stainless Steel

This chapter discusses how AM thermal history regulates the multiscale microstructure of austenitic stainless steels and how these microstructural features determine service performance. Precise microstructural control is essential for achieving reliable service performance in additively manufactured austenitic stainless steels. Additive manufacturing, characterized by rapid solidification and complex thermal cycling, often produces epitaxially grown columnar grains with strong crystallographic textures. It also generates multiscale microstructural features, such as residual δ-ferrite, high-density dislocation cell structures, elemental segregation, and, in some cases, nanoscale oxides or inclusions. These multiscale features collectively affect strength, ductility, fatigue resistance, corrosion behavior, hydrogen-assisted cracking susceptibility, and mechanical anisotropy. Accordingly, recent studies have focused on regulating the solidification mode, crystallographic orientation, grain-boundary character distribution, and post-processing-induced microstructural evolution to optimize the performance of AM austenitic stainless steels. These strategies are closely related to the defect formation and environment-assisted degradation mechanisms discussed in Section 2, because pores, cracks, residual stresses, δ-ferrite, and hydrogen trapping behavior are strongly governed by local solidification conditions and subsequent microstructural evolution.

### 3.1. Solidification Mode

**Figure 7:**
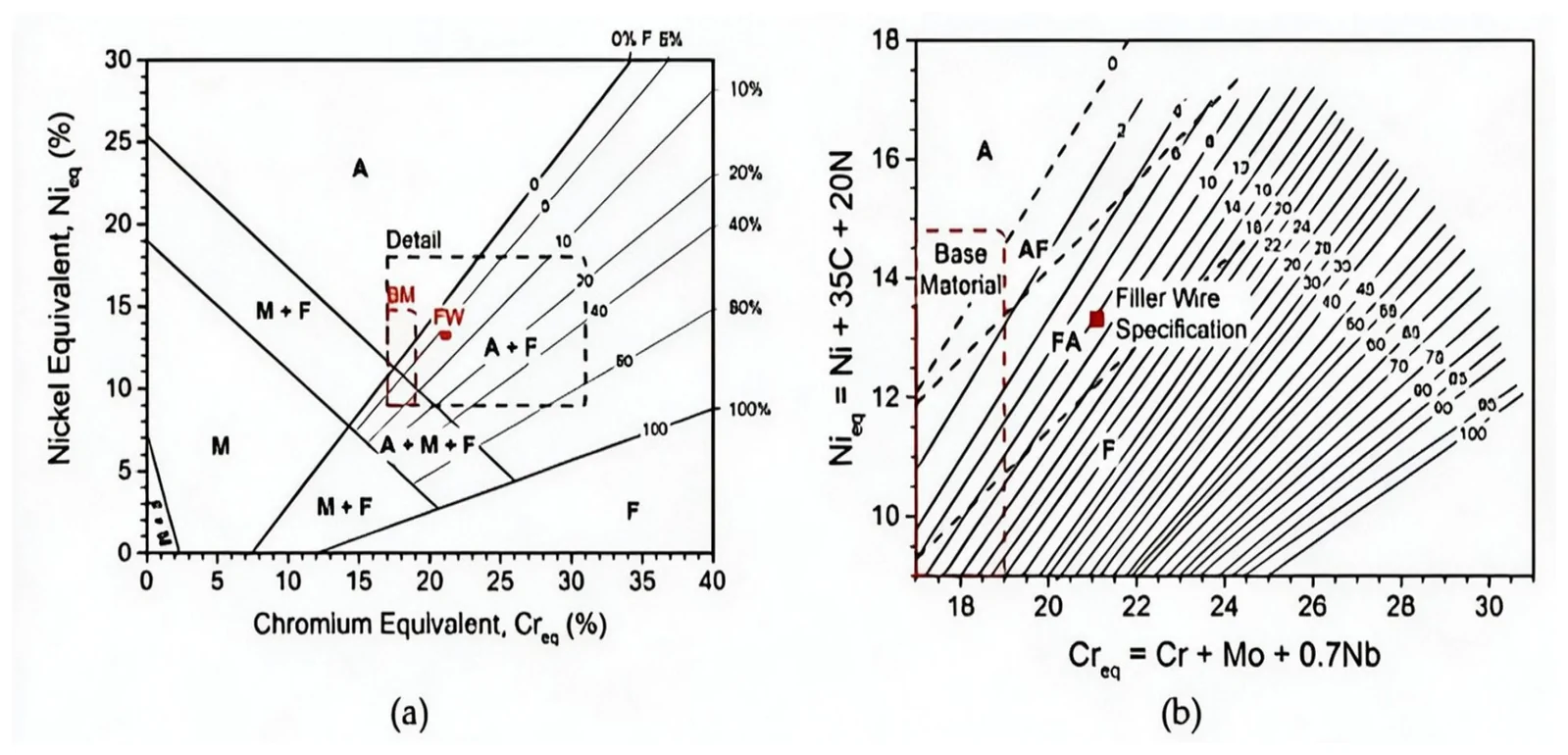
Prediction and solidification behavior of austenitic stainless steels: microstructure prediction based on chromium and nickel equivalents using Schaeffler and WRC diagrams [142,143]. (**a**) Reprinted from [142], Pilhagen J. A literature review of the stainless steel 21-6-9 and its potential for sandwich nozzles[J]. 2007; (**b**) Reprinted from [143], Kotecki, D.J. WRC-1992 constitution diagram for stainless steel weld metals: A modification of the WRC-1988 diagram. Weld. J. 1992, 71, 171–178.

The solidification mode plays a central role in linking the thermal conditions of additive manufacturing, including heat input, thermal gradient, solidification rate, cooling rate, and melt-pool dynamics, to the resulting phase constitution and microstructural morphology. It largely determines whether grains grow epitaxially along the heat-flow direction as columnar grains or transform into fine equiaxed grains through heterogeneous nucleation, depending on the interplay among the thermal gradient, solidification rate, and nucleation undercooling. In general, a high thermal-gradient-to-solidification-rate ratio (G/R) favors planar or columnar dendritic growth, whereas a lower G/R promotes constitutional undercooling and equiaxed grain formation. Meanwhile, the cooling rate, often approximated by G × R, controls the dendrite arm spacing, cellular substructure size, and solute segregation scale. Therefore, the solidification mode in AM austenitic stainless steels should be interpreted as the combined result of alloy chemistry and local melt-pool thermal conditions. Chemical composition is a key factor controlling the intrinsic solidification path, but in additive manufacturing the actual solidification mode is also strongly affected by non-equilibrium cooling, melt-pool geometry, repeated remelting, thermal cycling, and local compositional redistribution. For austenitic stainless steels, the solidification tendency can be semi-quantitatively estimated using chromium and nickel equivalents, Cr_eq_ and Ni_eq_ as described by the Schaeffler diagram [142] and the WRC-1992 diagram [143] (Figure 7). These diagrams are useful for estimating the nominal ferrite/austenite balance and possible solidification path under conventional welding-like conditions, but their applicability to AM must be treated with caution because of rapid solidification and repeated thermal cycling. By analyzing the Cr_eq_ and Ni_eq_ values, the alloy can be located within a nominal microstructural region, enabling a preliminary estimation of the expected ferrite/austenite balance based on the chemical composition of the built component. The Cr_eq_ and Ni_eq_ values are calculated using empirical coefficients that represent the ferrite- and austenite-stabilizing effects of individual alloying elements, respectively.

Cr_eq_ = Cr + Mo + 0.7 Nb (wt%) (1)

Ni_eq_ = Ni + 35 C + 20 N + 0.25 Cu (wt%) (2)

The WRC-1992 formulation is generally applicable to stainless steel systems with Cr_eq_ values of 8–26 wt.% and Ni_eq_ values of 2–18 wt.%. However, in additive manufacturing, where cooling rates may reach 10^5^–10^7^ K·s^−1^, the actual solidification path can deviate from these predictions because of non-equilibrium solute redistribution, solute trapping, repeated remelting, and shielding-gas-induced compositional changes. Therefore, Schaeffler and WRC-1992 diagrams based on Cr_eq_/Ni_eq_ should be regarded as first-order screening tools rather than deterministic predictors of phase constitution in AM components. When the actual phase constitution, δ-ferrite fraction, morphology, distribution, and crystallographic characteristics must be confirmed, EBSD, XRD, and TEM should be employed, combined with EDS/EPMA for local composition analysis. XRD rapidly provides bulk-averaged phase constitution, relative δ/γ content, texture, and residual stress; EBSD maps grain morphology, orientation, grain-boundary character, and the location and morphology of δ-ferrite; TEM reveals cellular dislocation structures, nanoscale oxides, precipitates, and the fine structure of δ/γ interfaces; and EDS/EPMA determines microsegregation between dendrite cores and interdendritic regions and the redistribution of Cr/Ni/Mo [37,104,144,145,146]. When the non-equilibrium solidification path, solute trapping, phase selection (A, F, or FA mode), and phase fractions must be predicted, CALPHAD-based calculations such as the Scheil solidification model or phase-field simulations should be employed, the former approximating rapid solidification with negligible back-diffusion and the latter reproducing multiphase cooperative growth and solute redistribution, thereby supporting composition design, process-window prediction, and cross-validation with measured phase constitution [144,147]. When the local thermal history of the melt pool must be quantitatively linked to the cooling rate, thermal gradient G, solidification rate R, and solute redistribution, and when the influence of process parameters on solidification microstructure, residual stress, and distortion must be predicted, thermal–fluid coupled simulations should be used; combined with CCT/TTP diagrams or Johnson–Mehl–Avrami-type kinetic models, they can further predict solid-state phase transformations during repeated multilayer heating [147,148,149]. These three categories of methods are complementary: CALPHAD and thermal–kinetic models provide predictions and process guidance, while EBSD/XRD/TEM provide experimental verification, thereby compensating for the limitation that Schaeffler and WRC-1992 diagrams can serve only as first-order estimates under AM conditions.

Variations in the Cr_eq_/Ni_eq_ ratio correspond to different solidification modes in austenitic stainless steels. For example, 316L stainless steel commonly solidifies in the ferrite–austenite (FA) mode, following L → L + δ → L + δ + γ → γ + δ, where a small amount of residual δ-ferrite may remain in the austenitic matrix. In contrast, 310 stainless steel, with a lower Cr_eq_/Ni_eq_ ratio, tends to solidify in the austenitic (A) or austenite–ferrite (AF) mode, following L → L + γ → γ or L → L + γ + δ → γ + δ. The presence of residual δ-ferrite has a dual effect on AM austenitic stainless steels. A small amount of δ-ferrite can reduce solidification cracking susceptibility by accommodating impurity segregation and interrupting continuous liquid films during terminal solidification. However, excessive or continuous δ-ferrite networks may deteriorate corrosion resistance, cryogenic toughness, and hydrogen embrittlement resistance, as discussed in Section 2. Therefore, controlling not only the volume fraction but also the morphology, distribution, and connectivity of δ-ferrite is essential. The predominantly austenitic solidification path and high Ni/Cr content of 310 stainless steel contribute to improved high-temperature phase stability, although its solidification cracking susceptibility and microsegregation behavior must also be considered. The δ-ferrite network can accommodate impurity elements such as S and P, thereby reducing their segregation tendency at austenite grain boundaries [50]. Moreover, δ-ferrite can interrupt the continuous growth of austenite grains and influence grain-boundary migration, thereby contributing to grain refinement [150,151]. However, compositional and electrochemical differences between δ-ferrite and the γ-austenite matrix can induce micro-galvanic coupling [152]. Excessive, inhomogeneous, or continuous δ-ferrite distribution, especially along grain boundaries, can promote localized corrosion and reduce resistance to stress-corrosion cracking and hydrogen-assisted cracking [153]. During heat treatment or service at 500–900 °C, brittle σ phase and M_23_C_6_ carbides may precipitate preferentially in or near δ-ferrite, compromising toughness and corrosion resistance. At cryogenic temperatures—for example, near −189 °C—the body-centered cubic structure of δ-ferrite can promote cleavage fracture, thereby significantly reducing impact toughness [147,150,154].

Overall, the net effect of δ-ferrite depends strongly on its volume fraction, morphology, distribution, and service environment: a moderate amount of discretely distributed δ-ferrite is beneficial for reducing solidification cracking susceptibility, whereas excessive or continuous networks degrade corrosion resistance, cryogenic toughness, and hydrogen-assisted cracking resistance through micro-galvanic coupling, cleavage fracture, and fast hydrogen diffusion pathways.

Shielding gases can alter the solidification mode by modifying the chemical composition of the melt pool and by affecting heat input, arc or laser–plasma stability, melt-pool convection, and defect formation. Common shielding gases include N_2_, He, Ar, or combinations thereof [11,155,156,157]. As reported by Shi et al., incorporating N_2_ into the shielding gas increases nitrogen uptake in the deposit, stabilizes austenite, suppresses δ-ferrite formation, promotes the development of cellular or columnar austenitic dendrites, and can reduce localized corrosion susceptibility [158,159]. This modification may shift the solidification mode from ferrite–austenite (FA) to austenite–ferrite (AF), or even toward a fully austenitic (A) mode. However, excessive nitrogen uptake can promote nitrogen-related porosity and significantly reduce fracture strain, thereby causing premature brittle fracture [160].

In arc-based additive manufacturing, the high ionization potential and thermal conductivity of He can increase arc voltage, energy density, and penetration depth. Enhanced melt-pool convection can refine cellular or columnar austenitic dendrites and reduce dendrite arm spacing [161]. Adding He to N_2_-containing shielding gases can partially restore fracture strain and mitigate nitrogen-induced embrittlement, possibly by improving melt-pool convection and reducing nitrogen-related pore retention [61,155]. Ar shielding provides a stable arc and effectively protects the melt pool from oxidation and atmospheric contamination [162]. Under pure Ar shielding, deposits often exhibit a duplex γ + δ structure, with vermicular δ-ferrite distributed within the austenitic matrix; this microstructure can provide good ductility and moderate strength when the δ-ferrite fraction and distribution are properly controlled [163]. Introducing a small amount of CO_2_, typically around 2.5 vol.%, into Ar shielding can reduce surface oxides, improve arc stability, and increase deposition efficiency. However, excessive CO_2_ can increase carbon pickup and promote Cr_23_C_6_ precipitation during exposure at 450–900 °C, leading to local chromium depletion and intergranular corrosion [164]. Zhong et al. [165] introduced an Ar–N_2_–He mixed shielding gas in laser–MIG hybrid additive manufacturing of 316L stainless steel. Compared with the conventional Ar–CO_2_ shielding system, the 80%Ar–10%N_2_–10%He mixture enabled in situ nitrogen alloying and improved the as-built properties without post-treatment, mainly because of enhanced austenite stability, refined cellular substructures, and reduced δ-ferrite content.

Overall, the solidification mode determines the initial phase constitution, dendritic morphology, and grain-growth mode, thereby establishing the foundation for microstructure control in AM austenitic stainless steels. During layer-by-layer deposition, epitaxial growth, competitive grain selection, remelting, and repeated thermal cycling further modify grain morphology and crystallographic orientation, leading to characteristic textures. These textures strongly influence anisotropic deformation, slip activation, crack propagation, corrosion behavior, hydrogen-assisted cracking susceptibility, and interfacial stability. Therefore, understanding solidification-mode control provides a necessary basis for discussing crystallographic orientation and texture evolution in the following section.

### 3.2. Crystallographic Orientation

Crystallographic orientation is critical in determining the performance of additively manufactured austenitic stainless steels by influencing the elastic modulus, activation of {111}<110> slip systems, interfacial characteristics, residual stress distribution, fatigue crack propagation, corrosion behavior, and hydrogen-assisted cracking susceptibility [33,161,166,167,168]. In FCC austenite, dendritic growth preferentially occurs along the <1> crystallographic direction. During AM solidification, grains whose <1> direction is closely aligned with the maximum thermal gradient have a competitive growth advantage and may overgrow less favorably oriented grains. Therefore, crystallographic texture in AM austenitic stainless steels is fundamentally governed by the coupling among epitaxial growth, competitive grain selection, melt-pool geometry, and local heat-flow direction.

In single-track multilayer additive manufacturing of cubic metals, particularly austenitic stainless steels, solidification is governed by a high thermal gradient G and rapid solidification rate R within the melt pool. These conditions promote epitaxial growth from previously solidified layers and competitive selection of grains whose <001> direction is aligned with the local heat-flow direction, leading to columnar grains with strong near-<001> or {100}-type build-direction textures [169,170,171,172,173,174]. In WAAM components, the apparent orientation distribution may appear diagonal in certain sections because the build-direction fiber texture is projected onto specific sampling or testing geometries, such as 45° diagonal specimens. Altering the welding torch angle—for example, from 5° to 15°—can modify melt-pool geometry and heat-flow direction, thereby promoting the development of preferred crystallographic orientations. Consequently, the texture-favored diagonal direction may exhibit a higher elastic modulus because of the orientation dependence of elastic properties. This texture, together with process-induced microstructural features such as residual δ-ferrite, dislocation cell structures, elemental segregation, and, in some cases, nanoscale oxides, constitutes a multiscale microstructure.

From a performance standpoint, a strong {100}-type texture can facilitate the activation of multiple {111}<110> slip systems under favorable loading orientations because of a relatively uniform Schmid factor distribution, thereby improving ductility and resistance to fatigue crack growth under certain conditions [73,173,175,176]. However, the beneficial effect of texture may be weakened or even reversed when it is coupled with elongated columnar grains, residual tensile stresses, lack-of-fusion defects, or δ-ferrite networks. Therefore, crystallographic texture should be evaluated together with defect population and microstructural heterogeneity, rather than as an isolated parameter. During deformation, initial orientations such as (113) and (114) may rotate toward orientations close to (13) and (199) as a result of slip-system activation and dislocation accumulation. At a strain of 60%, the deformation texture can become dominated by orientations near (115) and (122) [175]. In particle- or carbide-reinforced stainless steel systems, crystallographic orientation can also affect interfacial bonding between the matrix and strengthening phases. For example, Bai et al. [168] reported that the Fe(111)/WC(1) interface exhibited the highest stability among the investigated configurations, indicating the importance of orientation relationship in particle-reinforced stainless steel composites.

To better understand the crystallographic nature of interfacial bonding and precipitation, the near-coincidence site lattice (NCS) model provides a useful framework for analyzing interfacial structures, orientation relationships, and habit planes between different crystal phases [177,178,179]. Gao et al. [179], investigated the co-precipitation behavior of carbides in high-alloy stainless steels. They found that M_23_C_6_ carbides maintain a cube–cube orientation relationship with the γ-austenite matrix. M_6_C carbides tend to nucleate heterogeneously at pre-existing M_23_C_6_/γ interfaces and inherit the associated {111} habit plane. This leads to a closely connected co-precipitation morphology and reveals the crystallographic basis for the synergistic precipitation of multiple carbides in high-alloy stainless steels. For AM austenitic stainless steels, such orientation relationships are relevant because rapid solidification and subsequent thermal cycling can promote nonequilibrium segregation and carbide precipitation at cell boundaries, grain boundaries, or δ/γ interfaces. These precipitate–matrix orientation relationships may influence local strengthening, corrosion resistance, and crack initiation behavior.

In multilayer and multipass additive manufacturing, scanning strategy influences laser–material interaction time, spatial heat accumulation, interlayer reheating, heat-flow direction, and the continuity of epitaxial grain growth. These factors modify the melt-pool morphology, thermal gradient G, solidification rate R, and cooling rate G × R, thereby controlling grain morphology and texture intensity [148,180]. Forooghi et al. reported that meander scanning—namely, continuous reciprocating scanning—achieved a high cooling rate of up to 4.85 × 10^6^ K·s^−1^ and produced a relatively shallow, conduction-dominated melt pool because of its efficient laser path. This scanning mode can promote [101,111] oriented texture components, fine equiaxed grains, and a higher fraction of high-angle grain boundaries, thereby improving microstructural homogeneity and potentially enhancing strength–toughness synergy. Nevertheless, the beneficial effect of high-angle grain boundaries depends on their character and network connectivity. Random high-angle grain boundaries may become preferential paths for intergranular corrosion or hydrogen-assisted cracking, whereas low-energy special boundaries, such as Σ3 twin boundaries, are generally more resistant to boundary degradation. This issue is further discussed in the following section on grain-boundary engineering. Conversely, stripe or chessboard scanning may promote transitional or keyhole-type melt pools when local heat accumulation and melt-pool overlap become significant. Under such conditions, the thermal-gradient direction may change, causing deviation of grain-growth direction and the formation of coarse columnar grains with a strong [111]-dominated texture. These scanning paths can also increase dislocation density and the fraction of low-angle grain boundaries, which may enhance strength but also increase mechanical anisotropy when coupled with strong columnar textures.

The incorporation of trace alloying elements such as B, Nb, and Ti can significantly influence solidification structure, grain morphology, and crystallographic orientation. These elements affect material properties mainly by altering constitutional undercooling, heterogeneous nucleation behavior, solute drag, particle pinning, and phase stability [181]. During solidification, boron (B) can segregate at the solid–liquid interface and form a solute-enriched boundary layer, increasing constitutional undercooling. This process can promote the columnar-to-equiaxed transition, refine grains, weaken crystallographic texture, and randomize grain orientations [182]. Similarly, titanium (Ti) can form heterogeneous nucleation particles, such as TiN, TiC, or Ti-rich oxides, which promote non-epitaxial nucleation, refine grains, and weaken texture [183]. Niobium (Nb) can inhibit grain-boundary migration, refine the microstructure, and modify texture through solute drag and Nb-rich carbide/carbonitride formation [184]. However, excessive Nb segregation or Laves phase formation may deteriorate ductility and corrosion resistance. These alloying additions modify solidification kinetics and interface stability by increasing non-epitaxial nucleation events and altering competitive grain growth, thereby weakening inherited epitaxial textures. Consequently, they regulate grain morphology, grain size, orientation distribution, and ultimately the isotropy of mechanical properties. The synergy between alloying-element design and process-parameter control can improve strength, toughness, and isotropy, providing an important strategy for designing high-performance AM austenitic stainless steels.

Texture describes the overall orientation distribution of grains, whereas grain boundaries characterize the misorientation relationship and interface structure between adjacent grains. Although they represent different hierarchical descriptors of microstructure, texture and grain-boundary character jointly influence dislocation transmission, crack propagation, corrosion paths, and hydrogen-assisted cracking. Therefore, controlling grain-growth direction or texture alone is insufficient for precise property optimization; coordinated regulation of texture, grain-boundary character distribution, and defect population is required. Accordingly, improving microstructural stability and service performance requires not only texture control but also grain-boundary engineering, particularly by increasing the fraction of low-energy special boundaries and reducing the connectivity of random high-angle grain-boundary networks.

### 3.3. Grain Boundary Engineering

Grain boundary engineering (GBE) aims to optimize the grain-boundary character distribution (GBCD) of austenitic stainless steels, traditionally through thermomechanical treatments such as deformation and annealing [11,185,186]. In additively manufactured austenitic stainless steels, GBE is also strongly influenced by rapid solidification, epitaxial grain growth, repeated thermal cycling, residual stresses, and high-density dislocation substructures. The primary goal of GBE is to increase the fraction of low-Σ coincident site lattice (CSL) boundaries, particularly low-energy Σ3 twin boundaries, while disrupting the connectivity of random high-angle grain-boundary networks. Such boundary-network optimization can improve intergranular corrosion resistance, fracture resistance, strength–ductility synergy, and resistance to hydrogen-assisted cracking [77,187,188]. This is particularly important for AM austenitic stainless steels because random high-angle grain boundaries and phase interfaces can act as preferential paths for intergranular corrosion, hydrogen segregation, and crack propagation, as discussed in Section 2. In contrast, coherent or near-coherent Σ3 twin boundaries generally exhibit lower boundary energy, reduced impurity segregation tendency, and higher resistance to boundary decohesion [117,118,127]. In additive manufacturing, rapid solidification and repeated thermal cycling provide unique opportunities for in situ grain-boundary modification, although the resulting GBCD is highly sensitive to melt-pool geometry, epitaxial growth, scanning strategy, and local thermal history. High-density dislocations and residual stresses introduced during AM provide stored energy for recovery and recrystallization during subsequent annealing, thereby promoting grain-boundary migration and the formation of annealing twins such as Σ3 boundaries [189]. In addition, adjusting AM parameters, such as heat input and scanning strategy, or introducing in situ alloying can modify the thermal gradient, solidification rate, cooling rate, and competitive grain-growth behavior, thereby influencing the solidification structure. Under appropriate processing and compositional conditions, these strategies can promote equiaxed grain formation, weaken inherited epitaxial textures, and increase the fraction of special grain boundaries [36]. Nevertheless, achieving controllable GBE during AM remains challenging. The GBCD is spatially heterogeneous because of local variations in melt-pool geometry, remelting depth, thermal gradient, cooling rate, and hatch overlap. Moreover, strong epitaxial growth may preserve columnar grains across multiple layers, limiting the formation of randomly oriented recrystallized grains and annealing twins. Therefore, in situ GBE usually needs to be combined with post-processing treatments to obtain a stable and favorable grain-boundary network. For instance, grain-refining elements such as Ti and Nb can promote the formation of second-phase particles, including Ti-rich oxides, TiN, TiC, NbC/NbN, and Nb-rich Laves phases, depending on the local oxygen, nitrogen, carbon, and niobium contents. These particles can act as heterogeneous nucleation sites to refine grains and restrict grain-boundary migration through Zener pinning, thereby altering the GBCD [190,191,192]. However, excessive or coarse particles, particularly brittle Laves phases, may become crack-initiation sites and deteriorate ductility or corrosion resistance.

**Figure 8:**
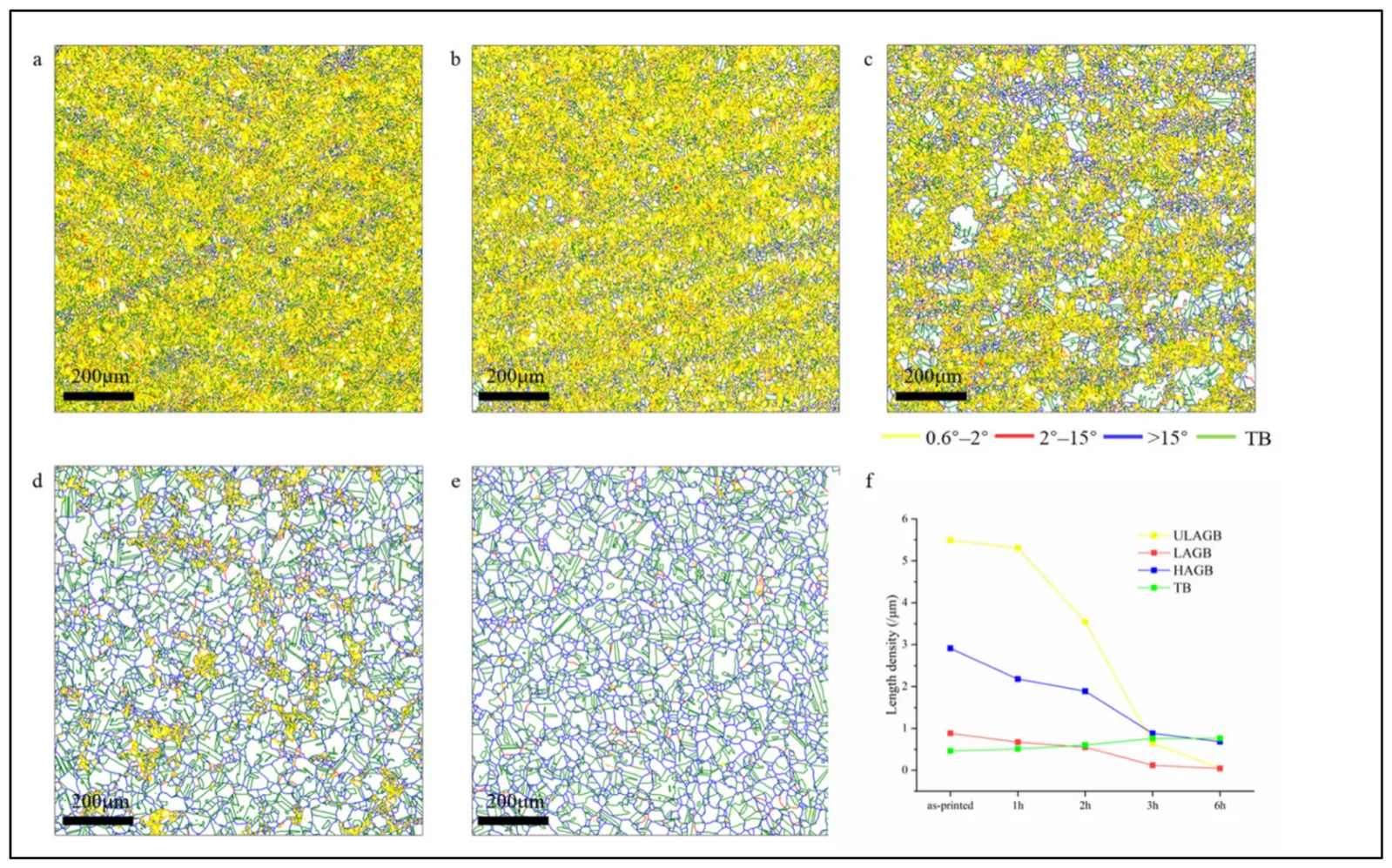
EBSD orientation maps of the SLM 316L stainless steel samples showing boundary misorientations in different angle ranges: (**a**) as-printed, and annealed at 1000 °C for (**b**) 1 h, (**c**) 2 h, (**d**) 3 h, and (**e**) 6 h; (**f**) variation in length density (LA) for the four ranges/types of boundaries analyzed [199]. Reprinted from [199], Wang W-Y et al., Effect of Heat Treatment on Microstructural Evolution in Additively Manufactured 316L Stainless Steel, *Metals*, published by MDPI, 2023 (CC BY 4.0).

The addition of rare-earth oxide particles, such as La_2_O_3_, can promote martensitic transformation and deformation twinning under cryogenic deformation conditions, leading to the formation of α′-martensite and an increased fraction of Σ3 twin boundaries [190]. However, many austenitic stainless steels, particularly Ni-rich systems, exhibit relatively high stacking fault energies, which suppress deformation twinning and reduce the probability of annealing-twin formation. This limitation makes it more difficult to construct low-Σ CSL boundary networks through conventional thermomechanical or annealing treatments [193,194]. To overcome these challenges, researchers have explored strategies that regulate stacking fault energy, phase stability, and thermal history. These strategies include co-adding Ti and Mn to balance phase constitution and regulate stacking fault energy [195], using liquid-metal-assisted LPBF to modify heat transfer and solidification behavior [196], and applying cryogenic treatment to promote deformation twinning or martensitic transformation [197]. These process innovations aim to tailor thermal history and post-treatment heat regimes to suppress unfavorable phases, such as σ phase and brittle Laves phases, while promoting recrystallization, annealing-twin formation, and low-Σ CSL boundary development [149,198], as shown in Figure 8.

Composition design for GBE can be classified according to its dominant mechanism: promoting heterogeneous nucleation, pinning grain-boundary migration, regulating stacking fault energy, stabilizing dislocation cell structures, and controlling segregation or precipitation at boundaries. Targeted composition design is also critical for GBE because alloying elements can regulate precipitation, elemental segregation, stacking fault energy, grain-boundary migration, and recrystallization behavior [200]. For example, Al addition can promote the formation of high-density nanoscale precipitates within austenite grains, which may stabilize dislocation cell-wall structures in AM austenitic stainless steels. This can provide precipitation strengthening while influencing grain-boundary migration, local strain accommodation, and interfacial stability. In addition to precipitation, elemental segregation and stacking fault energy regulation are key mechanisms by which alloying elements affect GBE and deformation behavior. Kong et al. [109] demonstrated that Mo addition promotes Mo segregation at dislocation cell boundaries in AM austenitic stainless steels. This segregation can enhance yield strength and pitting corrosion resistance, but it may also increase stacking fault energy, suppress deformation twinning, and reduce work-hardening capacity and ductility. Conversely, Si addition can lower stacking fault energy, although it may also promote Si/Mn-rich oxide formation and affect local corrosion resistance. The reduced stacking fault energy promotes a transition from wavy or multiple slip to planar slip and twinning-assisted deformation, inducing stacking faults and nanotwins at the early stage of deformation [144]. As a result, work-hardening capacity and uniform elongation can be improved, enabling a more favorable strength–ductility synergy.

The corrosion resistance of grain-boundary-engineered (GBE) additively manufactured austenitic stainless steels is governed not only by the total fraction of low-Σ coincident-site-lattice (CSL) boundaries, but also by their network connectivity, the degree of deviation from the ideal CSL relationship, and the topology of triple junctions. Low-Σ CSL boundaries, especially coherent Σ3 twin boundaries, exhibit low boundary energy and a weak tendency for impurity segregation and carbide precipitation, and are therefore more resistant to intergranular corrosion than random high-angle grain boundaries (HAGBs); however, their beneficial effect is maximized only when these boundaries form a connected network that effectively interrupts the percolation path of random HAGBs, whereas isolated low-Σ boundaries embedded in a continuous random-boundary network provide limited protection [36,185,194]. The degree of deviation from the ideal CSL relationship is equally important, since within the Brandon criterion or the stricter Palumbo–Aust tolerance a boundary retains its low-energy character, while beyond this threshold the boundary energy and excess free volume increase, promoting segregation, precipitation, and localized corrosion, so that coherent or near-coherent Σ3 boundaries are superior to incoherent Σ3 boundaries, which behave increasingly like random boundaries [185,187]. At the mesoscale, triple junctions formed entirely by low-Σ CSL boundaries are structurally ordered and low-energy sites that are resistant to corrosion initiation, whereas triple junctions containing random HAGBs act as preferential initiation sites because of their excess free volume and local electrochemical heterogeneity [185,194]. Taken together, at the network scale, a well-connected CSL network combined with low-Σ CSL triple junctions is the most favorable configuration, although it should be noted that this assessment is established primarily on GBE and corrosion studies of wrought austenitic stainless steels, whereas direct experimental evidence for AM alloys remains limited because rapid solidification, solute segregation, and residual stresses modify boundary structure; when low-Σ CSL boundaries are sufficiently interconnected and uniformly distributed, they can interrupt the percolation path of the random HAGB network, thereby suppressing intergranular corrosion propagation and improving local strain compatibility. Overall, GBE provides a bridge between solidification-induced grain structures, crystallographic texture, and service performance, and although AM offers opportunities for tailoring the grain-boundary character distribution (GBCD) through thermal cycling, scan-path design, and in situ alloying, post-processing remains essential for promoting recrystallization, annealing-twin formation, residual stress relief, and boundary-network optimization, the influence of which on these microstructural features is discussed in the following section [36,185,194].

### 3.4. Influence of Post-Processing

Although optimization of heat input, scanning strategy, and alloy composition can improve grain morphology, crystallographic texture, and grain-boundary character distribution, AM components in the as-built condition typically exhibit relatively high surface roughness, which is considerably greater than that of conventionally machined or polished surfaces. For LPBF, the as-built surface roughness Ra is typically in the range of approximately 5–20 μm [82,201,202], whereas WAAM and DED can exhibit considerably higher roughness, reaching several tens to hundreds of micrometers, depending on process parameters, build orientation, and surface location [10,11,203]. Such rough as-built surfaces, together with residual stresses, residual pores, and lack-of-fusion defects, often necessitate post-processing to meet service requirements. This indicates that in situ microstructural and defect control during fabrication alone is often insufficient, and post-processing treatments are essential for bridging the gap between the as-built microstructure and service-state performance. These treatments play a vital role in homogenizing microstructures, relieving residual stresses, closing internal defects, and improving surface integrity [11,89].

Different post-processing routes affect AM components through distinct mechanisms, including defect closure, residual stress relaxation, recrystallization, precipitation control, surface smoothing, work hardening, and surface chemistry modification. For AM austenitic stainless steels, post-processing methods can be broadly classified into thermal treatments, hot isostatic pressing, mechanical surface treatments, chemical/electrochemical surface finishing, cryogenic treatments, and hybrid processing routes. Thermal treatments mainly regulate residual stress, segregation, δ-ferrite stability, precipitation, and recrystallization behavior. HIP is primarily used to close internal pores and improve fatigue resistance. Surface treatments mainly improve surface integrity and near-surface residual stress states, whereas cryogenic treatments regulate deformation substructures, nanotwins, and phase stability. Surface modification and finishing methods, including surface mechanical attrition treatment [204,205], high-pressure torsion [206,207], shot peening [205,208,209,210,211], and polishing [212,213,214], have been used to improve the surface integrity and near-surface microstructure of AM components. These methods can reduce surface roughness, generate gradient refined structures, and introduce compressive residual stresses and work-hardened layers, thereby improving resistance to fatigue crack initiation, localized corrosion, and surface-assisted hydrogen ingress [205,208,209]. This is particularly important because surface-connected pores, partially melted particles, and high roughness can act as preferential sites for fatigue crack initiation, pit nucleation, and hydrogen entry. However, excessive surface plastic deformation may also increase dislocation density and near-surface hydrogen trapping sites, which should be considered for hydrogen-containing service environments.

Thermal treatments, such as stress-relief annealing, solution annealing, and recrystallization annealing, significantly influence the evolution of non-equilibrium solidification structures, including dislocation cell structures, elemental segregation, residual δ-ferrite, precipitates, and recrystallized grains. Hot isostatic pressing further promotes defect closure through the combined effects of high temperature and isostatic pressure [215,216,217,218,219,220,221,222,223,224,225,226,227]. Key microstructural features, including columnar or interlayer equiaxed grain morphology, fine cellular substructures, dislocation networks, segregation patterns, and nanoscale oxide inclusions, evolve systematically with heat-treatment temperature and holding time [199,224,225].

**Figure 9:**
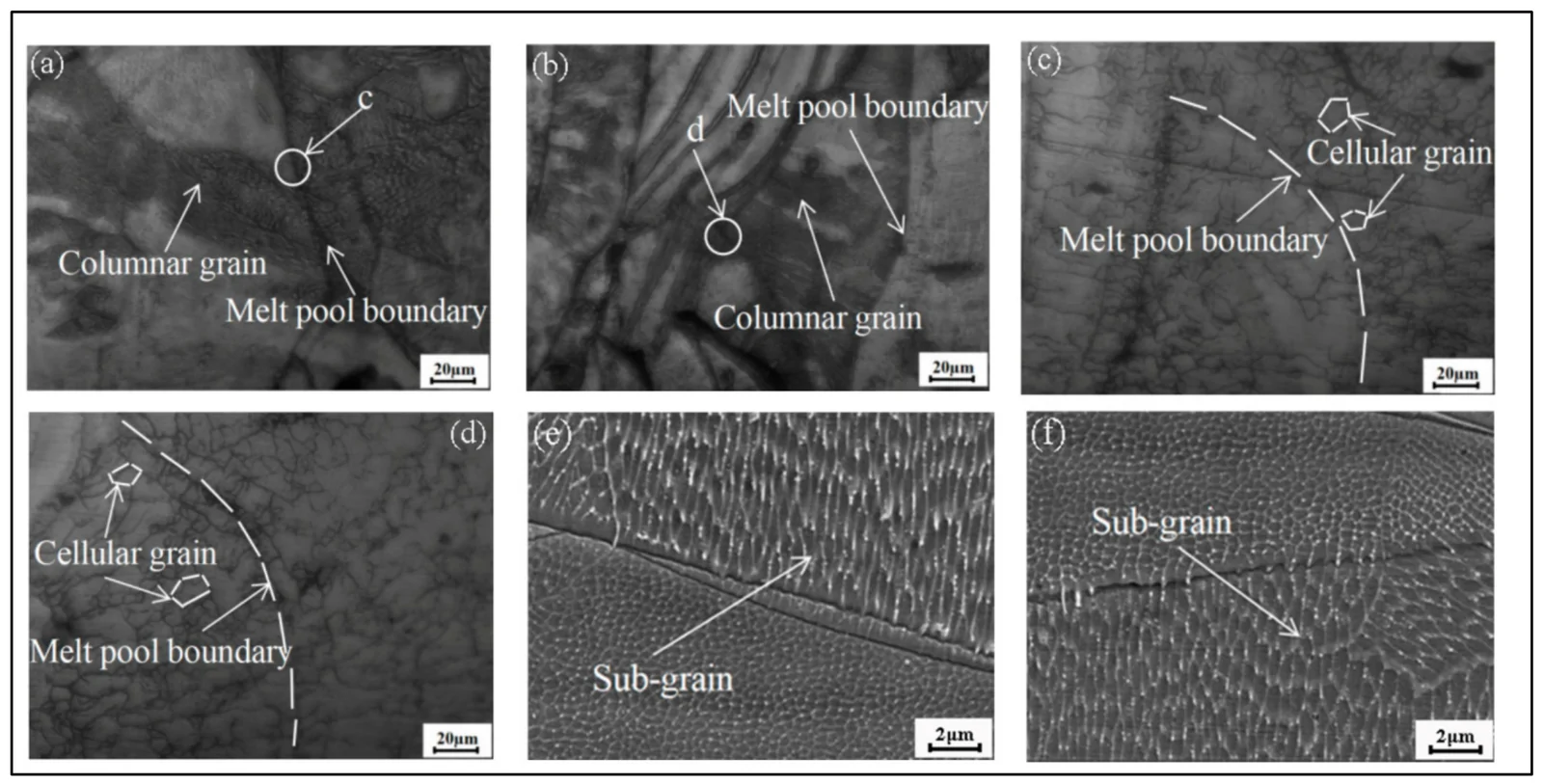
Microstructure of the PBF-LB/M 316L stainless steel specimens under different heat-treatment conditions: (**a**) as-built; (**b**) HT1 (500 °C); (**c**) HT2 (800 °C); (**d**) HT3 (1100 °C); (**e**) magnified view of region c; and (**f**) magnified view of region d [230]. Reprinted from [230], Zhou Y et al., The Effects of Heat Treatment Temperatures on the Properties of 316L Stainless Steel Produced via Laser Powder Bed Fusion, *Materials*, published by MDPI, 2025 (CC BY 4.0).

Stress-relief annealing can reduce residual stresses while partially retaining the as-built dislocation cell structure and associated strengthening. Solution or recrystallization annealing can homogenize segregation, dissolve or reduce δ-ferrite and secondary phases, and promote equiaxed recrystallized grains and annealing twins. However, excessive temperature or holding time may cause grain coarsening, loss of dislocation strengthening, and precipitation of deleterious phases, indicating that heat-treatment windows must be optimized according to the target balance between strength, ductility, corrosion resistance, and hydrogen embrittlement resistance. Figure 8 and Figure 9 demonstrate that at lower temperatures (below 800 °C), the cellular structure remains relatively stable, with minimal change in grain size. However, when temperatures reach 800 °C and above, the cellular structure coarsens and gradually disintegrates, influenced by grain boundary diffusion rates and initial microstructure. Additionally, nano-oxides coarsen significantly with increased temperature and time, especially under high-temperature, long-duration treatments [226]. As temperatures exceed 1100 °C, dislocation tangles in the cellular structure are gradually eliminated, facilitating the recrystallization of austenite grains. This process promotes the transformation from columnar to equiaxed grains and is accompanied by grain growth [221,227,228]. At this stage, the coarsening of oxides is primarily driven by lattice diffusion [226,229]. Furthermore, heat treatment affects the formation and evolution of secondary phases, such as δ-ferrite, σ phase, and carbides. The type, fraction, and distribution of phases such as residual δ-ferrite, σ phase, M_23_C_6_ carbides, and Laves phases are governed by the cooling rate during AM and by subsequent heat-treatment temperature and time [226,230].

Variations in the initial microstructure and defect state produced by different AM processes strongly affect recrystallization behavior, dislocation-structure evolution, defect healing, and deformation mechanisms during subsequent post-processing. For instance, laser powder bed fusion, characterized by high cooling rates, typically produces fine austenitic grains with high dislocation density, lattice strain, cellular substructures, elemental microsegregation, and high residual stresses [229,231]. After hot isostatic pressing, pore closure occurs together with recovery and, in some cases, recrystallization because of the high stored energy in the as-built microstructure. This reduces dislocation density and may shift the dominant deformation mode from twinning-assisted deformation toward dislocation slip, depending on grain size, stacking fault energy, and post-processing parameters [232,233], HIP is particularly effective for closed or nearly closed internal pores, improving density and fatigue performance. However, it is less effective for surface-connected defects and may induce grain growth or precipitation changes. Therefore, HIP is often more effective when combined with surface finishing or subsequent heat treatment. In contrast, arc-based additive manufacturing, especially WAAM, involves high heat input, strong thermal accumulation, and layer-by-layer deposition, resulting in coarse columnar grains that grow epitaxially along the build direction. These grains may contain high-density dislocations, cellular substructures, residual δ-ferrite, and significant segregation of elements such as Cr and Mo at cell walls because of non-equilibrium solidification and repeated thermal cycling [150,234].

During subsequent heat treatment or plastic deformation, coarse columnar grain boundaries and segregated cell walls can promote heterogeneous recrystallization and local deformation, thereby prolonging the recrystallization incubation period. These segregated cell walls can pin dislocations, provide dislocation strengthening, and enhance strain-rate sensitivity during the early stages of deformation. They may also increase the critical stress required for deformation twinning, thereby delaying twin activation and maintaining dislocation-slip-dominated deformation over a wider strain range. Sonis et al. [150] reported that WAAM 316L exhibits millimeter-scale columnar grains, a vermicular δ-ferrite network with a volume fraction of approximately 5–8%, and relatively large, low-density Si/Mn-rich oxide particles with an average size of about 500 nm and a number density of approximately 10^10^ m^−2^. After stress-relief annealing at 600 °C, the δ-ferrite network and large oxide particles are largely retained, indicating that low-temperature annealing is insufficient to substantially modify the as-deposited microstructure. High-temperature recrystallization treatment at 1200 °C can dissolve δ-ferrite and transform columnar grains into equiaxed grains. Because of their relatively large size and high stability, the oxide particles show limited coarsening or grain-boundary segregation during this treatment. This treatment mitigates low-temperature brittleness and increases impact toughness at room temperature and −193 °C by 60% and 475%, respectively, accompanied by a transition from brittle fracture to fully ductile fracture.

Recent advances in temperature-based post-processing have expanded from conventional stress relief and solution treatment to high-temperature recrystallization and cryogenic treatment, enabling broader control of microstructure and mechanical properties in AM austenitic stainless steels. Cryogenic treatment has attracted increasing attention because it can regulate dislocation configurations, promote nanotwin formation, and, in metastable alloys, induce martensitic transformation [235,236]. Applying liquid nitrogen in situ during arc-based additive manufacturing can disrupt the epitaxial growth of columnar grains. The altered thermal boundary conditions promote a more randomly oriented equiaxed grain structure, thereby reducing mechanical anisotropy and improving elastic-modulus uniformity [237]. Furthermore, short-term cryogenic treatment of LPBF 316L stainless steel can introduce high-density nanotwins without significantly compromising ductility. These nanotwin boundaries act as effective barriers to dislocation motion, increasing the yield strength by more than 12%. However, the strengthening effect is highly sensitive to treatment duration. When the duration exceeds 15 min, material softening may occur because of reduced dislocation density, degradation of cellular substructures, and nanotwin coarsening [238]. Therefore, cryogenic treatment should be regarded as a narrow-window microstructure-tuning strategy rather than a universally strengthening treatment. Its effectiveness depends on alloy stability, initial dislocation density, cellular substructure integrity, and treatment duration. To further exploit the potential of cryogenic treatment, researchers have combined it with surface severe plastic deformation methods, such as high-energy shot peening. Nanotwins introduced by cryogenic pretreatment, together with modified lattice spacing, can enhance the response to subsequent high-energy shot peening. This combined treatment increases the thickness of the severely plastically deformed layer to 91 μm, producing substantial strength gains while maintaining good ductility [209]. These studies demonstrate that cryogenic treatment, whether used as a post-treatment method or an in situ control strategy, provides a low-temperature microstructure-modification pathway distinct from conventional high-temperature heat treatments. It can strengthen and toughen AM austenitic stainless steels by inducing nanotwins, regulating dislocation configurations, and optimizing grain morphology, thereby serving as a complementary strategy to conventional heat treatments.

Overall, post-processing should not be regarded as a universal performance-enhancement step; its effectiveness depends strongly on the initial microstructure, defect population, residual stress state, surface condition, and intended service environment. For AM austenitic stainless steels, a more effective strategy is to design post-processing routes tailored to specific service requirements. This requires selecting appropriate post-processing routes with the objectives of defect mitigation, microstructural optimization, surface-integrity improvement, and service-environment adaptation, particularly for fatigue-, corrosion-, or hydrogen-assisted cracking-dominated applications.

## 4. Research on the Comprehensive Properties of Additively Manufactured Austenitic Stainless Steel

As discussed in Section 3, the solidification mode in additively manufactured austenitic stainless steels largely determines the initial grain morphology, phase constitution, and solute redistribution. Crystallographic texture governs orientation-dependent deformation and property anisotropy, whereas the grain-boundary character distribution affects local strain compatibility, interfacial stability, corrosion paths, crack propagation, and hydrogen-assisted cracking susceptibility. Post-processing also contributes substantially to defect mitigation, residual stress relief, surface-integrity improvement, and microstructural optimization. These microstructure–defect–surface control strategies are strongly interconnected and collectively form the foundation for optimizing the service performance of additively manufactured austenitic stainless steels. However, the ultimate aim of microstructural control is not merely to obtain a specific microstructure, but to improve the overall service performance of AM austenitic stainless steels under realistic corrosion, wear, fatigue, and coupled environmental–mechanical conditions. Therefore, a systematic analysis of the relationships among microstructural characteristics, defect states, surface integrity, and service-relevant properties is essential. In this chapter, corrosion resistance, wear resistance, and fatigue performance are discussed as representative service properties. Particular attention is paid to how AM-induced nonequilibrium microstructures, residual stresses, surface roughness, pores, lack-of-fusion defects, δ-ferrite, solute segregation, texture, and grain-boundary networks jointly determine performance.

It should be noted that the corrosion, wear, and fatigue data discussed in this chapter were obtained under different testing conditions. Environmental, loading, and specimen-related factors can all significantly affect the measured response and thus limit direct comparison across studies. Quantitative values reported in different studies are therefore compared with caution and are used primarily to illustrate trends and underlying mechanisms rather than to establish intrinsic, universally comparable performance indices.

### 4.1. Corrosion Behavior and Resistance

The corrosion behavior of AM austenitic stainless steels is governed by the stability, compactness, and self-repairing ability of the passive film, which are controlled by alloy composition, microsegregation, phase constitution, grain-boundary character, residual stress, and surface condition. Unlike conventionally processed stainless steels, AM materials often contain nonequilibrium cellular substructures, residual δ-ferrite, heterogeneous solute distribution, high dislocation density, surface roughness, and process-induced defects. These features can either enhance passivation by refining microstructures and promoting Cr diffusion or deteriorate corrosion resistance by creating micro-galvanic couples, Cr-depleted regions, stress-assisted dissolution sites, and preferential chloride-penetration paths.

#### 4.1.1. Influence of Process Characteristics

The corrosion resistance of additively manufactured austenitic stainless steels varies significantly with AM process, corrosion medium, surface condition, defect population, residual stress state, and post-processing history. Fine grains, high-density dislocations, cellular substructures, and residual δ-ferrite produced during AM can enhance passivation in some environments, but they may also promote localized corrosion when associated with severe segregation, micro-galvanic coupling, residual tensile stress, or unstable passive-film formation. For instance, under specific high-temperature air and salt-environment conditions, WAAM-produced 316L stainless steel has been reported to exhibit better corrosion resistance than conventionally forged counterparts. This improvement has been attributed to fine dendritic substructures and vermicular δ-ferrite, which promote outward Cr diffusion and the formation of a continuous Cr-rich oxide/passive layer [239]. However, such comparisons should not be generalized solely according to process type. The corrosion response is controlled by the process-induced thermal history and its consequences for surface roughness, porosity, residual stress, segregation, phase constitution, and passive-film stability. Similarly, in high-temperature and high-pressure water environments, LPBF-HIP and PM-HIP 316L demonstrate improved resistance to stress corrosion cracking due to fine grains and dispersed nano-oxides that inhibit strain localization [240]. Conversely, structural inhomogeneity introduced during additive manufacturing can weaken corrosion resistance. For example, 316L produced by LMD shows significantly lower corrosion resistance than traditional materials in molten chloride salts. This is because δ-ferrite segregates at grain boundaries, creating preferential corrosion paths [241]. Additionally, regions with higher residual δ-ferrite content in WAAM 340 stainless steel exhibit more pronounced oxidation weight gain in molten salt environments, indicating that local microstructural differences can impact corrosion response [45]. These findings suggest that compositional segregation and structural inhomogeneity, arising from differences in heat input, cooling rate, remelting, and thermal cycling, can alter local oxidation behavior and passive-film stability, thereby increasing the likelihood of localized corrosion.

During AM, energy input affects melt-pool temperature fields, element redistribution, possible element evaporation or pickup, solidification behavior, and microsegregation, thereby influencing the corrosion resistance of stainless steels [152,242]. Variations in corrosion resistance among different AM processes primarily originate from differences in heat input, cooling rate, melt-pool stability, defect formation, surface roughness, residual stress, and post-processing response. For instance, in L-PBF, the high cooling rate promotes fine grain formation, high-density grain-boundary networks, nanoscale oxide inclusions, and strong residual stress fields. In chloride-containing environments, these features can increase pitting-initiation sensitivity, particularly when nanoscale oxides, residual tensile stresses, and surface-connected defects act synergistically [240,243,244,245]. In contrast, WAAM generally exhibits slower cooling rates and may retain more δ-ferrite networks, which can contribute to good uniform corrosion resistance in some neutral environments when the δ/γ phase distribution is stable and electrochemically balanced. However, insufficient control of the interlayer temperature, particularly above 196 °C, can promote Cr_23_C_6_ precipitation along grain boundaries, leading to Cr-depleted regions and markedly deteriorating intergranular corrosion resistance [38,75,246,247,248]. These examples indicate that the corrosion response of AM austenitic stainless steels is governed by the combined effects of cooling rate, heat accumulation, residual stress, phase constitution, and defect distribution rather than by the AM process category alone. Therefore, no general conclusion can be made regarding the intrinsic superiority of corrosion resistance among different AM processes; instead, corrosion performance should be evaluated by considering process parameters, defect state, surface condition, post-processing history, and service medium. Excessive heat input can generate local high-temperature regions, causing redistribution, oxidation, or loss of key corrosion-resistant elements in the melt-pool and reheated zones. Depletion of corrosion-resistant elements such as Cr and Mo, together with changes in Ni-controlled phase stability, weakens passive-film stability and pitting resistance, thereby increasing localized corrosion susceptibility [249,250,251]. In addition, high-temperature processing may promote oxygen pickup and oxide inclusion formation, which can provide preferential sites for corrosion initiation [100,252].

**Figure 10:**
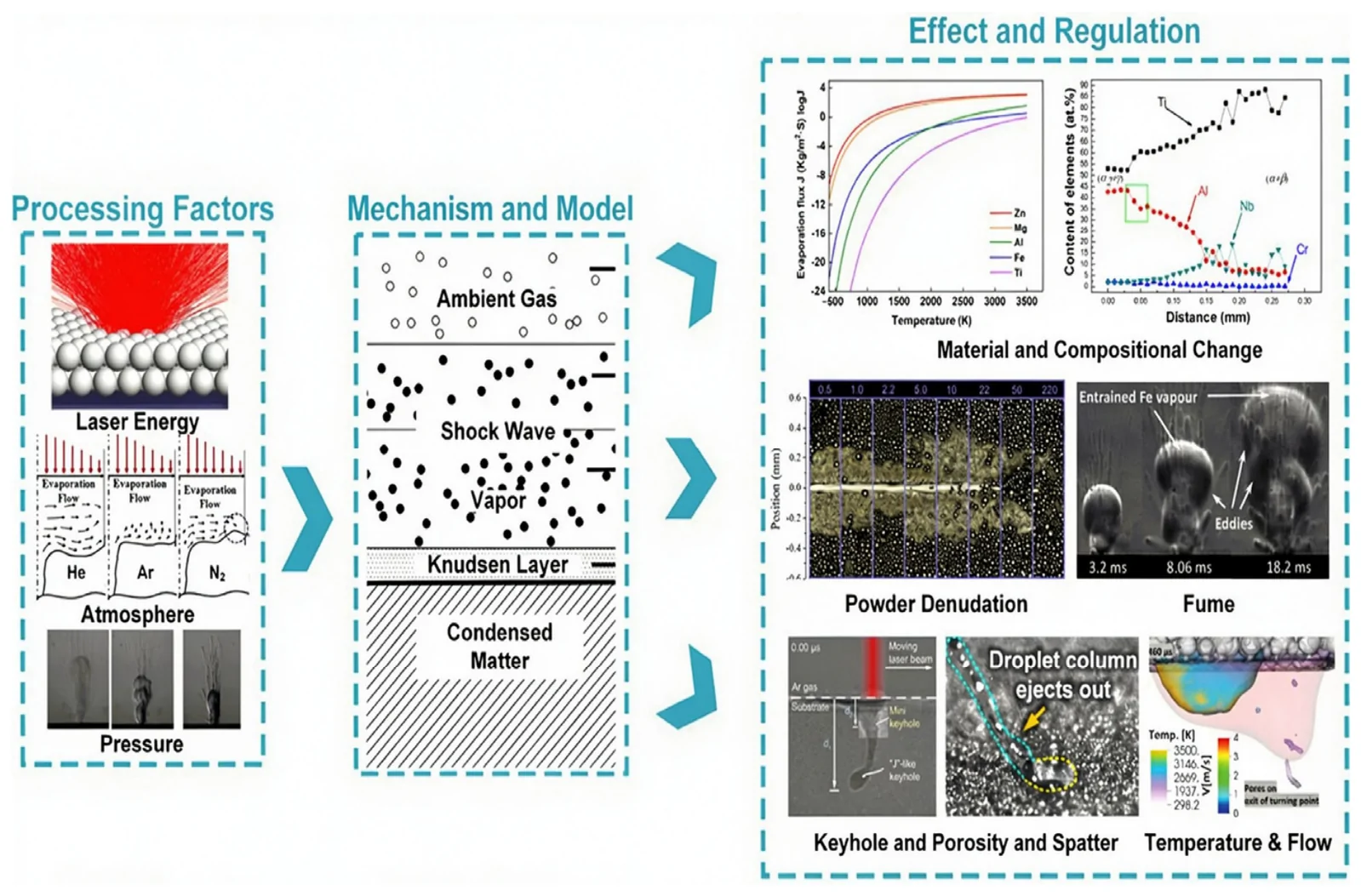
Schematic illustration of metal evaporation during laser powder bed fusion [253]. Reprinted from [253], Liu J et al., Metal vaporization and its influence during laser powder bed fusion process, *Materials & Design*, published by Elsevier, 2022 (CC BY 4.0).

Metal evaporation is a critical phenomenon in high-temperature processes. As shown in Figure 10, elements with low boiling points, such as manganese, chromium, and nickel, tend to volatilize, altering the material’s chemical composition and impacting its corrosion resistance and mechanical strength [65,253,254,255,256,257,258,259,260,261]. Furthermore, compositional changes, particularly the chromium-to-nickel equivalent ratio (Cr_eq_/Ni_eq_), strongly affect the solidification mode and thus the δ-ferrite fraction, segregation behavior, and susceptibility to Cr-depleted regions. Figure 7 indicates that, under conventional welding-like conditions, a Cr_eq_/Ni_eq_ ≥ 1.51 may correspond to a possible AF-to-FA transition [142,143]. In AM, this trend should be verified against measured δ-ferrite fraction and phase constitution. In the literature, such diagram-based predictions are generally corroborated by EBSD/XRD/TEM characterization rather than used as standalone proof. In the reported case, this transition reduced elemental segregation and mitigated intergranular Cr-depleted zones, changing the corrosion morphology from continuous grooves to dispersed pits and decreasing the corrosion rate by approximately 13% under the reported nitric acid test conditions [257]. These effects should be attributed to the measured changes in solidification microstructure, segregation, and Cr-depleted zones under those specific conditions, rather than to the nominal Cr_eq_/Ni_eq_ threshold alone. Therefore, the fundamental difference in corrosion resistance among AM processes does not arise from the process category itself, but from the combined effects of thermal history on composition, microstructure, defect state, surface integrity, and residual stress. Accordingly, process-related corrosion behavior should be interpreted through a coupled framework involving heat input, cooling rate, melt-pool stability, surface quality, defect characteristics, and post-processing state rather than through a direct comparison of AM process names.

#### 4.1.2. Influence of Microstructure

The corrosion resistance of additively manufactured austenitic stainless steels is strongly influenced by solidification microstructure, phase constitution, crystallographic orientation, grain-boundary character distribution, and passive-film stability. These factors collectively affect corrosion behavior by controlling the solidification path, passive-film formation and stability, elemental redistribution, micro-galvanic coupling, and preferential corrosion pathways. At the microstructural level, the solidification mode largely determines phase constitution, grain morphology, and segregation characteristics. Under conventional solidification conditions, a relatively low Cr_eq_/Ni_eq_ ratio tends to favor a predominantly austenitic solidification mode, and this tendency is often used as a first-order guide. In AM, the actual phase constitution and δ-ferrite fraction should be corroborated by EBSD/XRD/TEM and local composition analysis because rapid solidification and solute trapping can shift the effective solidification path. Rapid cooling can produce fine cellular or columnar substructures that reduce the segregation length scale and mitigate the formation of Cr-depleted zones [258]. If δ-ferrite formed during FA solidification has a composition close to that of the γ-austenite matrix, δ/γ micro-galvanic coupling can be reduced.

However, excessive or continuous δ-ferrite can increase δ/γ interfacial area, disrupt passive-film continuity, and promote micro-galvanic corrosion; when accompanied by σ phase or Cr/Mo segregation, it may further accelerate groove-like localized corrosion [257,259]. Increasing the processing atmosphere pressure can shift the solidification mode from AF toward a fully austenitic A mode, reduce the δ-ferrite content to below 0.1%, and suppress Cr-depleted zones and interphase galvanic effects. Higher nitrogen content can stabilize austenite, promote rapid regeneration of Cr_2_O_3_-rich passive films, and enhance repassivation by nitrogen-containing corrosion products or oxides [159,260]. A gradient solidification structure, evolving from planar growth near the bottom region to equiaxed dendritic growth near the top region, can reduce Cr and Mo segregation and promote a more uniform honeycomb-like subgrain structure [76]. Regarding grain morphology and size, as reported by Shi et al., increasing the fraction of low-angle grain boundaries (LAGBs) and reducing grain-boundary energy can enhance passive-film compactness and stability [261]. For instance, Zhang et al. [262] refined grains and increased the fraction of low-Σ CSL boundaries by introducing heterogeneous nucleation cores, such as TiC particles. Xu et al. [112] and Zhang et al. [263] also improved corrosion resistance by refining grains and increasing the LAGB fraction, which reduced local interfacial energy and promoted more uniform passive-film formation.

In terms of crystallographic orientation, the (111) plane can promote Cr_2_O_3_ nucleation and growth because of its high atomic packing density and relatively low surface energy. Adjusting the (111)/(200) intensity ratio—for example, increasing it to 2.25 through solution treatment—can modify surface crystallographic orientation and increase the Cr_2_O_3_ content in the passive film [264]. Adding TiC nanoparticles or generating TiC in situ can reduce the texture index, weaken crystallographic anisotropy, and promote more uniform passive-film coverage across different grain orientations [112,262]. In N08926 coatings prepared by laser cladding, a preferred (196) orientation has also been reported to enhance passive-film compactness and stability, indicating that the beneficial orientation may depend on alloy system, surface state, and corrosion medium [76]. From the perspective of grain-boundary engineering, Σ3 twin boundaries and LAGBs possess relatively low interfacial energy and ordered atomic arrangements. They can suppress Cr segregation and Cr-depleted boundary formation, disrupt the connectivity of random high-angle grain-boundary networks, impede Cl^−^ diffusion pathways, and reduce micro-galvanic corrosion. Adding TiC nanoparticles increases the fraction of low-Σ CSL boundaries, thereby reducing grain-boundary energy and corrosion susceptibility [262]. In situ synthesis of a (Ti,Nb)C-reinforced composite coating by laser cladding increased the LAGB fraction from 55.0% to 87.7%, thereby improving passive-film stability and corrosion resistance [263]. Overall, microstructural control improves corrosion resistance not through a single factor, but through the coordinated regulation of phase balance, segregation scale, crystallographic orientation, grain-boundary network connectivity, and passive-film chemistry.

#### 4.1.3. Influence of Alloying Elements and Processing Atmosphere

Table 3 summarizes the effects of key alloying elements on the corrosion behavior of additively manufactured austenitic stainless steels. Under AM, the actual retained composition can deviate from the nominal value because of selective evaporation (e.g., Mn, Cr), atmospheric N/O/C pickup, and local segregation. These deviations change the effective γ/δ balance and can make the nominal Cr_eq_/Nie_q_ ratio a poor predictor of local corrosion susceptibility. For example, Mo and Cr increase ferrite stability, whereas N, Ni, and Mn stabilize austenite; these effects modify the γ/δ phase balance, segregation behavior, and local corrosion susceptibility [264,265]. In particular, nitrogen can mitigate pitting corrosion through interstitial solid-solution strengthening and passivation-related mechanisms. As reported by Shi et al., nitrogen has been shown to delay the initiation and propagation of pitting corrosion. This effect is mainly attributed to local acid neutralization by NH_4_^+^ formation, enhanced passive-film formation, nitrate-related inhibition, and electrostatic repulsion against Cl^−^ adsorption or penetration [158,266].

In terms of corrosion regulation, alloying elements affect additively manufactured austenitic stainless steels through three primary mechanisms. First, elements such as Cr, Mo, and N are essential for passive-film formation, stabilization, and repassivation, which are critical for pitting corrosion resistance [29]. Second, Ni, Mn, Cr, and N influence the solidification mode and extent of segregation by regulating austenite/ferrite stability, thereby indirectly affecting local corrosion susceptibility [265,273]. Third, during high-temperature AM processes, element evaporation, redistribution, and oxidation can alter the actual composition, leading to discrepancies between the designed composition and the as-built or service-state composition [249,274]. Therefore, corrosion analysis should consider not only nominal alloy ratios but also the actual composition after AM thermal cycling and subsequent exposure, including local depletion/enrichment of Cr, Mo, Ni etc. Cr_eq_/Ni_eq_ calculated from nominal feedstock composition should be regarded as a design-level descriptor; when used to explain corrosion or phase selection, it should be recalculated from local measured compositions and coupled with phase-fraction data and EBSD/XRD/TEM characterization. Computational modeling can support this evaluation by predicting melt-pool behavior, element loss, and process windows that minimize evaporation-induced compositional deviations. For instance, melt-pool simulations help clarify the loss mechanisms of volatile elements during AM [275]. Overall, the beneficial or detrimental role of alloying elements depends on their actual retained content, spatial distribution, interaction with C/O/N/S impurities, and influence on passive-film chemistry and phase stability.

#### 4.1.4. Influence of Inclusions and Reinforcing Particles

The influence of inclusions and reinforcing particles on the corrosion resistance of additively manufactured austenitic stainless steels has been widely investigated. Harmful inclusions can damage the passive film by creating local Cr-depleted regions, passive-film discontinuities, and electrochemical heterogeneity around the inclusion/matrix interface [174,276]. This degradation is associated with spontaneous inclusion dissolution and electrochemical potential differences between inclusions and the surrounding matrix [277]. These differences promote local galvanic interactions and accelerate localized corrosion. In AM 316L components, inclusions can act as cathodic sites and form galvanic couples with the anodic γ-austenite matrix, thereby accelerating matrix dissolution [278]. Silica-containing inclusions can promote the formation of Si-rich compounds that hinder passive-film formation, increase metal dissolution, and facilitate pit growth [242]. In contrast to harmful inclusions, controlled reinforcing additives such as CNTs [276], CeO_2_ [278], and TiC [279] have been explored to improve the corrosion resistance of AM 316L stainless steel. Uniformly distributed particles can serve as heterogeneous nucleation sites, refine grains, and promote the formation of a more continuous and uniform passive film. Such a distribution can reduce average dislocation density, lower stored energy, and decrease electrochemical heterogeneity [280]. Nanoparticles can also alter melt-pool dynamics and solidification behavior. Under appropriate conditions, particles with high thermal stability can facilitate grain refinement, increase the fraction of low-Σ CSL boundaries, and reduce impurity aggregation, thereby decreasing intergranular corrosion susceptibility [262]. Certain Ti-containing nanoparticles can improve passive-film chemistry by increasing the Cr_2_O_3_/Cr(OH)_2_ ratio, suppressing Cr-carbide precipitation, and reducing point-defect density, thereby making the film less permeable to aggressive ions. During corrosion, local dissolution and hydrolysis can generate corrosion products that deposit at passive-film defects or early pitting sites, forming hydrated oxide colloids that fill defects and delay pit propagation [65,281]. Moreover, the passive film and nanoparticles may carry surface charges, thereby altering the charge distribution at the passive film/solution interface and influencing aggressive-ion adsorption [282].

Excessive particle addition can lead to agglomeration within the matrix, forming large cathodic regions that couple galvanically with the adjacent anodic matrix. This increases the local anodic current density, initiates severe micro-galvanic corrosion, and rapidly degrades the passive film [262]. Additionally, poor interfacial bonding between particles and the matrix can generate micropores and microcracks, which act as channels for corrosive media [235]. Therefore, the effect of inclusions and reinforcing particles on corrosion resistance is highly dependent on their size, distribution, chemical stability, interfacial bonding, and electrochemical compatibility with the matrix.

#### 4.1.5. Influence of Post-Processing

Post-processing is essential for regulating the corrosion resistance of additively manufactured austenitic stainless steels. Appropriate heat treatment can weaken or eliminate melt-pool boundary features and homogenize microsegregation, whereas surface strengthening or finishing can reduce roughness and modify residual stress states, thereby improving passive-film stability. Conversely, poorly controlled post-processing parameters may promote deleterious precipitation, such as Cr_23_C_6_ carbides or σ phase, and may also expose or redistribute pre-existing inclusions such as MnS. These changes increase electrochemical heterogeneity in the matrix and at grain boundaries, ultimately reducing corrosion resistance [36,217,225,283,284,285]. Therefore, the overall effect depends on the selected post-processing route, initial microstructure, defect state, surface condition, and service environment.

Surface treatments, including laser shock processing, electron-beam surface modification, mechanical polishing, electrochemical polishing, laser remelting, and shot peening, can reduce corrosion current density by decreasing surface roughness, removing partially melted particles, eliminating surface-connected pores, and introducing compressive residual stress [154,286,287,288]. Moderate near-surface work hardening may further improve passive-film stability, whereas excessive dislocation density or microstrain may have the opposite effect. In situ rolling, shot peening, and ultrasonic rolling can also decrease corrosion current density (icorr) by refining grains, increasing the LAGB fraction, disrupting dendritic structures, and promoting Cr-oxide enrichment and a higher O^2−^/OH^−^ ratio in the passive film. However, excessive surface strengthening or severe plastic deformation may introduce excessive dislocation density and strain-induced martensite, decreasing passive-film stability and increasing defect density [261,289,290,291]. It should be noted that in reported corrosion studies of AM austenitic stainless steels, surface roughness, partially melted particles, surface-connected pores, and surface finish have rarely been treated as independent variables. Most electrochemical and pitting tests are performed after grinding specimens to 2000 grit and polishing, thereby eliminating the influence of surface roughness and surface-connected defects and attributing the corrosion response primarily to microstructure, phase constitution, segregation, and residual stress [242,245,259]. Heat-treatment studies likewise generally use polished specimens and focus on precipitates, δ-ferrite dissolution, and grain-boundary characteristics [145,194,285,292,293]. Consequently, the corrosion resistance reported in the literature typically represents a “polished-state” or “near-surface defect-free state,” rather than the actual surface condition of AM components. For AM components without surface treatment, surface roughness increases the real surface area and promotes chloride accumulation; partially melted particles can form crevices and cause compositional heterogeneity with the matrix; and surface-connected pores provide penetration pathways for aggressive media, all of which may accelerate pitting, crevice corrosion, and stress corrosion cracking [154,249,259,268]. Therefore, the influence of surface integrity should be considered separately when evaluating the service reliability of actual components.

Heat treatments, particularly solution treatment, regulate corrosion resistance by modifying microsegregation, precipitate distribution, δ-ferrite stability, residual stress, and defect distribution [216,285,294,295]. Cr/Mo-rich precipitates, such as σ phase and M_23_C_6_ carbides, can dissolve into the matrix during high-temperature solution treatment, followed by rapid cooling to form a supersaturated solid solution. This process increases the concentration of corrosion-resistant elements in the matrix and promotes the formation of a protective Cr/Mo-enriched passive film, thereby improving resistance to localized corrosion. In addition, post-processing-induced changes in crystallographic texture, especially an increased (111)/(200) intensity ratio, can facilitate the formation of a dense and stable passive film. The treatment can also reduce the detrimental effect of MnS inclusions by modifying their distribution or interfacial chemistry, thereby decreasing their tendency to act as pitting-initiation sites [264,295]. At 900 and 1000 °C, extending the solution-treatment time from 1 to 4 h has been reported to gradually improve corrosion resistance under specific testing conditions. This improvement is mainly attributed to the dissolution of Cr/Mo-rich precipitates, homogenization of alloying elements in the matrix, and an increased (111)/(196) intensity ratio, which together promote the formation of a Cr_2_O_3_-rich passive film [145,264]. At 1050 °C, solution treatment can induce grain coarsening, reduce the fraction of random high-angle grain boundaries, increase Σ3 twin boundaries, and further dissolve σ phase. Inclusions may transform from MnO–SiO_2_–Cr_2_O_3_ complex oxides to more stable SiO_2_-rich inclusions, accompanied by minor MnS precipitation. These changes can increase passive-film thickness and stability, enrich MoO_3_ species, and thereby improve pitting resistance and corrosion impedance [289,292,296]. However, solution treatment above 1050 °C may provide only transient benefits in composition homogenization and passivation, particularly in acidic environments, if the holding time is not properly controlled. In chloride-containing environments, prolonged treatment from 1 to 4 h may instead reduce pitting resistance by promoting MnS precipitation, coarsening, or exposure at susceptible sites [264,293]. In addition, improper thermal exposure, particularly within the sensitization range of 500–900 °C, may promote carbide precipitation or harmful intermetallic phases such as σ phase along grain boundaries, thereby increasing susceptibility to intergranular corrosion and pitting [146,190,289,294]. Therefore, the heat-treatment window should be optimized according to alloy composition, initial AM microstructure, inclusion characteristics, and corrosion medium, rather than being selected solely on the basis of temperature or holding time. Overall, post-processing improves corrosion resistance only when defect mitigation, residual stress relief, surface integrity, phase stability, and passive-film chemistry are simultaneously optimized. Conversely, inappropriate post-processing may introduce sensitization, excessive deformation defects, or unstable phases, thereby accelerating localized corrosion.

### 4.2. Analysis of Wear Performance

The wear performance of additively manufactured austenitic stainless steels is governed by the combined effects of hardness, ductility, surface integrity, residual stress, porosity, reinforcement distribution, crystallographic texture, grain-boundary structure, and tribo-oxide film stability. Compared with conventionally processed counterparts, AM materials exhibit nonequilibrium cellular structures, solute segregation, retained δ-ferrite, process-induced defects, and high surface roughness, all of which can alter the transition among adhesive wear, abrasive wear, oxidative wear, delamination wear, and three-body wear. Therefore, the wear response should be interpreted through a coupled framework involving process characteristics, microstructure, temperature, inclusions or reinforcements, and post-processing.

#### 4.2.1. Influence of Process Characteristics

Austenitic stainless steels exhibit good ductility and toughness, but their relatively low hardness, together with AM-induced surface roughness and defects, can limit wear resistance. During sliding wear, the passive or tribo-oxide film may exhibit poor adhesion to the substrate; its repeated rupture and detachment can promote a transition from adhesive wear to combined adhesive–abrasive wear. This reduces service reliability in severe sliding, fretting, or particle-containing wear environments [297,298]. Traditional strategies for improving wear resistance, such as introducing hard particles including TiC or WC or applying heat treatment, often suffer from uneven particle distribution, weak particle/matrix interfacial bonding, and limited control over phase evolution or composition. These issues may introduce pores and cracks or cause substrate degradation, such as Cr loss, which can compromise both wear resistance and corrosion resistance [299,300,301,302]. Additive manufacturing provides a flexible route for wear-resistant material design by enabling control of process parameters, such as interlayer cooling time and scanning speed, and by allowing in situ reaction-based reinforcement formation [303,304,305,306,307]. For example, the in situ TiC can be generated from graphene and titanium powder during DED, producing uniformly distributed nano/microscale reinforcement phases. The combined effects of uniformly distributed reinforcements and grain refinement reduced the wear volume by 78.98% under the reported sliding-wear conditions [112]. In WAAM, introducing a high-Cr SS303 layer can increase the δ-ferrite fraction in the interfacial region and improve wear resistance by strengthening the interface and modifying phase distribution [308]. Extending the interlayer cooling time can reduce thermal accumulation, promote grain refinement, retain an appropriate amount of δ-ferrite, and thereby improve wear performance [309]. In material extrusion, trace B addition can form high-hardness Cr–B- and Mo–B-enriched grain-boundary phases. These phases can bond strongly with the matrix and resist plastic deformation during wear [310]. These AM strategies can mitigate some limitations of traditional methods by refining grains, regulating phase distribution, introducing strengthening phases, and improving interfacial bonding. They provide a flexible approach for designing wear-resistant components, provided that defect formation, surface quality, reinforcement distribution, and interfacial stability are properly controlled. Therefore, process-related wear behavior should be evaluated not only by hardness improvement but also by the combined evolution of surface roughness, porosity, residual stress, reinforcement/matrix interfaces, and tribo-oxide film stability.

#### 4.2.2. Influence of Microstructure

Microstructural anisotropy significantly influences wear performance by controlling the direction-dependent response of AM materials to contact stresses and sliding loads. As a result, wear mechanisms, wear rates, and wear-track morphologies can exhibit strong directional dependence [306,311]. Specifically, preferred grain orientation, or texture, affects slip-system activation, plastic deformation, and debris formation under sliding contact. Columnar grains and strong build-direction texture in AM austenitic stainless steels may promote anisotropic plowing and delamination, whereas equiaxed grains and weakened texture can improve the uniformity of load transfer during sliding. Grain-boundary character is also important because high-energy random boundaries may act as preferential sites for crack initiation and debris detachment, whereas low-energy boundaries can improve resistance to subsurface damage. Surface crystallographic orientation can affect susceptibility to plastic deformation and micro-cutting during sliding. For instance, grains with low Taylor-factor orientations can undergo easier directional shearing, leading to the formation of long, flaky wear debris. Additionally, boundary and substructural features, such as high-angle boundary density and cellular substructures, influence dislocation motion and crack propagation. A lower grain-boundary density can concentrate deformation within grains, promote long-range slip bands, and generate characteristic wear-debris morphologies [311]. A high-density dislocation-cell structure can strengthen the material and increase resistance to plastic deformation, although it may also raise frictional energy dissipation during sliding [312,313]. Recent advances in multiscale surface metrology further suggest that anisotropic surface topographies produced by directional finishing (abrasive-belt grinding) interact with liquid media and lubricants across discrete geometric scales, from microroughness to waviness, thereby influencing contact-line pinning and lubricant entrapment [314]. This scale-dependent perspective offers a useful framework for interpreting the direction-dependent wear behavior of AM austenitic stainless steels, where columnar grains and build-direction texture create inherently anisotropic surface geometries. Ultimately, these effects arise from the directional coupling between microstructural characteristics, including grain size, orientation, grain boundaries, and dislocation distribution, and the mechanical–thermal loads generated during frictional contact. Therefore, microstructure-sensitive wear behavior should be interpreted by linking crystallographic orientation, grain-boundary network, substructure stability, and debris evolution rather than by considering hardness alone.

#### 4.2.3. Influence of Temperature

The effect of temperature on the wear performance of additively manufactured austenitic stainless steels is complex and non-monotonic. This complexity arises because temperature strongly affects tribo-oxide film formation, surface softening, phase transformation, debris behavior, and the dominant wear mechanism [315,316]. In addition to service temperature, thermal control during AM, particularly interlayer cooling, is important for wear resistance. Lower interlayer temperatures and faster cooling rates can refine the microstructure, promote finer grains, retain an appropriate amount of δ-ferrite, and increase hardness and strength. These changes can result in a lower friction coefficient, reduced wear rate, and shallower wear tracks [317,318]. At low to moderate wear temperatures, approximately around 196 °C, surface oxides such as Fe_2_O_3_, Fe_3_O_4_, and Cr_2_O_3_ can form a protective glaze-like tribo-oxide layer. In metastable austenitic compositions, strain- or temperature-assisted martensitic transformation may also occur and contribute to a temporary reduction in friction coefficient and wear rate through a mixed oxidative–abrasive wear mechanism [315,316]. However, when the temperature increases from around 196 °C to 296–393 °C, pronounced thermal softening may occur, leading to decreased hardness and yield strength. Consequently, the protective oxide layer may crack and spall because of weakened adhesion, and the detached oxide fragments can exacerbate three-body abrasive wear. The softened matrix becomes more susceptible to adhesive transfer, severe plastic deformation, and delamination, shifting the dominant mechanism toward adhesive/plastic-deformation wear and markedly increasing the wear rate [33,319]. Thus, a moderate temperature increase may enhance wear resistance by promoting protective tribo-oxide formation, surface work hardening, or beneficial phase transformation. However, exceeding a critical temperature may reduce the load-bearing capacity of the matrix and destabilize the protective tribo-layer, thereby deteriorating wear performance. Therefore, the temperature dependence of wear resistance should be evaluated by distinguishing AM thermal history from service-temperature effects and by identifying the transition temperature at which protective tribo-films change from beneficial to unstable.

#### 4.2.4. Influence of Inclusions and Reinforcing Particles

Incorporating nanoscale ceramic particles can enhance mechanical properties when they are uniformly dispersed and well bonded to the matrix, because they act as barriers to dislocation movement and increase hardness. These nanoparticles can also serve as heterogeneous nucleation sites, inhibit grain growth, and refine the grain structure, thereby improving resistance to plastic deformation during wear [202,320]. Das et al. [321] demonstrated that graphene enhances matrix hardness through grain refinement and interfacial strengthening, while also forming a solid lubricating film on the counter-sliding surface during sliding, thereby reducing adhesive and abrasive wear. For instance, Liu et al. [322] utilized spherical WC particles as reinforcing phases to increase hardness and improve resistance to plastic deformation during sliding wear. After LPBF fabrication and aging at 800 °C, numerous submicron M_23_C_6_ carbides with sizes of 100–393 nm formed.

The material exhibited improved strength, toughness, and wear resistance owing to the synergistic effects of fine-grain strengthening, particle reinforcement, and precipitation hardening. Additionally, Karan et al. [304] fabricated WC/TiC dual nanocomposite reinforced 316L stainless steel using additive manufacturing. These specimens showed reduced plastic deformation and material loss during wear, mainly due to grain refinement, high dislocation density, and dislocation pinning by hard ceramic particles. Gain et al. [113] prepared a TiB_2_-reinforced SS316L nanocomposite and observed ultrafine grains beneath the worn surface, which formed through dynamic recrystallization induced by frictional heating and elastoplastic deformation. The combined effects of dynamic recrystallization-induced ultrafine grains and uniformly dispersed TiB_2_ nanoparticles reduced material removal and deformation under high-load conditions. However, excessive nanoscale ceramic particles can introduce chemical and structural defects, such as particle clusters, pores, and oxide-rich inclusions, thereby altering surface mechanical properties and wear mechanisms [110,243].

With increasing ceramic content, Marangoni convection in the melt pool can promote the agglomeration of nanoscale particles into microscale clusters, leading to stress concentration, crack formation, and degraded wear resistance [323]. Pores, as material discontinuities, can also cause stress concentration and act as crack-initiation sites under repeated contact or sliding loads, leading to local spalling and reduced wear resistance [298]. Oxide-rich inclusions, such as Si-, Mn-, and Cr-rich oxides, can act as hard protrusions during sliding and promote abrasive wear. These inclusions can plow and scratch the surface through two-body abrasion; once fractured or detached, they generate hard debris that promotes three-body abrasive wear. Their brittleness accelerates fragmentation and peeling, shifting the dominant mechanism from adhesive wear toward abrasive wear and degrading the overall tribological performance of the friction pair [110,324,325]. Therefore, the role of inclusions and reinforcing particles in wear resistance depends on their hardness, size, distribution, fracture resistance, interfacial bonding, and electrochemical or chemical stability with the matrix. Uniformly distributed reinforcements can improve load-bearing capacity, whereas brittle, agglomerated, or poorly bonded particles can become sources of cracks and third-body abrasives.

#### 4.2.5. Influence of Post-Processing

Post-processing treatments can play an important role in improving the wear performance of AM austenitic stainless steels [326,327]. Surface treatments directly modify surface morphology and near-surface microstructure, improving wear resistance by reducing roughness, introducing compressive residual stress, and forming strengthening phases or modified layers [287,328,329,330,331]. These mechanisms reduce micro-cutting and plowing, suppress contact-fatigue crack initiation, and increase near-surface load-bearing capacity through hard coatings or strengthened layers [331,332]. For instance, friction stir processing (FSP) uses frictional heat and severe plastic deformation to promote austenite stabilization or transformation, refine grains, close pores, and increase material density to 99.97%, thereby improving wear resistance [333]. Similarly, ultrasonic nanocrystalline surface modification (UNSM) can produce a nanocrystalline surface layer, high dislocation density, strain-induced martensite, and carbide precipitation through severe surface plastic deformation, thereby increasing surface hardness and wear resistance [332,334]. Premixed abrasive water jet (PAWJ) treatment and laser shock peening (LSP) can strengthen and smooth the surface with limited thermal effects, thereby improving surface integrity and wear resistance [335,336]. These surface-dominated treatments mainly improve wear resistance by modifying the near-surface stress state and roughness, whereas thermal treatments primarily act through phase and microstructural evolution. In contrast, heat treatment modifies the microstructure, phase constitution, residual stress, and hardness of the bulk material or surface layer, thereby affecting wear performance [298,337,338]. For example, under appropriate atmospheric and thermal conditions, heat treatment at 600 and 850 °C can promote the formation of stable oxide layers such as Cr_2_O_3_ and Fe_2_O_3_, reducing the wear rate and friction coefficient [339].

As reported by Vinothraj and Xavier, suitable heat treatment can adjust hardness, toughness, and residual stress, promote austenite twin formation, and regulate carbide distribution, thereby improving surface hardness and wear resistance [340,341]. However, excessively high temperatures may decrease microhardness through thermal softening or microstructural coarsening, thereby reducing wear resistance. In practical applications, hybrid post-processing strategies that combine heat treatment with surface modification are commonly adopted. Heat treatment can improve coating/substrate bonding and coating toughness in coated or cladded systems. Subsequent surface treatments, such as laser remelting and electrochemical polishing, can further reduce surface defects, smooth the surface, and promote the formation of a stable surface layer, producing synergistic improvements in wear resistance compared with single treatments, Nevertheless, post-processing parameters must be carefully optimized because excessive surface deformation, inappropriate heat input, or poorly bonded coatings may introduce cracks, delamination, tensile residual stress, or brittle phases that reduce wear resistance. Therefore, wear-oriented post-processing should be designed according to the dominant wear mechanism, surface integrity requirement, and service temperature [224,342,343].

### 4.3. Fatigue Fracture Analysis

The fatigue fracture behavior of additively manufactured austenitic stainless steels is governed by the coupled effects of defect population, surface integrity, residual stress, microstructural anisotropy, crystallographic texture, grain-boundary characteristics, post-processing state, and service environment. Compared with wrought counterparts, AM materials contain process-induced features such as lack-of-fusion defects, gas pores, unmelted particles, melt-pool boundaries, cellular substructures, solute segregation, and residual tensile stresses, which can accelerate crack initiation and alter crack-propagation paths. Therefore, fatigue performance should be interpreted through an integrated framework linking processing, microstructure, defects, loading direction, and environmental conditions.

#### 4.3.1. Characteristics of Fatigue Fracture

Under cyclic loading, fatigue failure generally proceeds through three stages: crack initiation, stable crack propagation, and final fracture. Crack initiation occurs when local plastic strain exceeds the deformation capacity of the material or when weak interfaces, such as inclusion/matrix interfaces and phase boundaries, separate under local stress concentration. In AM components, these initiation sites are typically stress-concentration regions, such as near-surface lack-of-fusion defects, pores, and inclusions. The corresponding fracture surfaces may exhibit fatigue striations, secondary cracks, tear ridges, and locally local quasi-cleavage facets [344,345,346]. Crack propagation is governed by the stress-intensity-factor range, crack-tip plasticity, residual stress state, and local microstructure. Final fracture occurs when the remaining load-bearing cross-section can no longer sustain the applied load [347].

**Figure 11:**
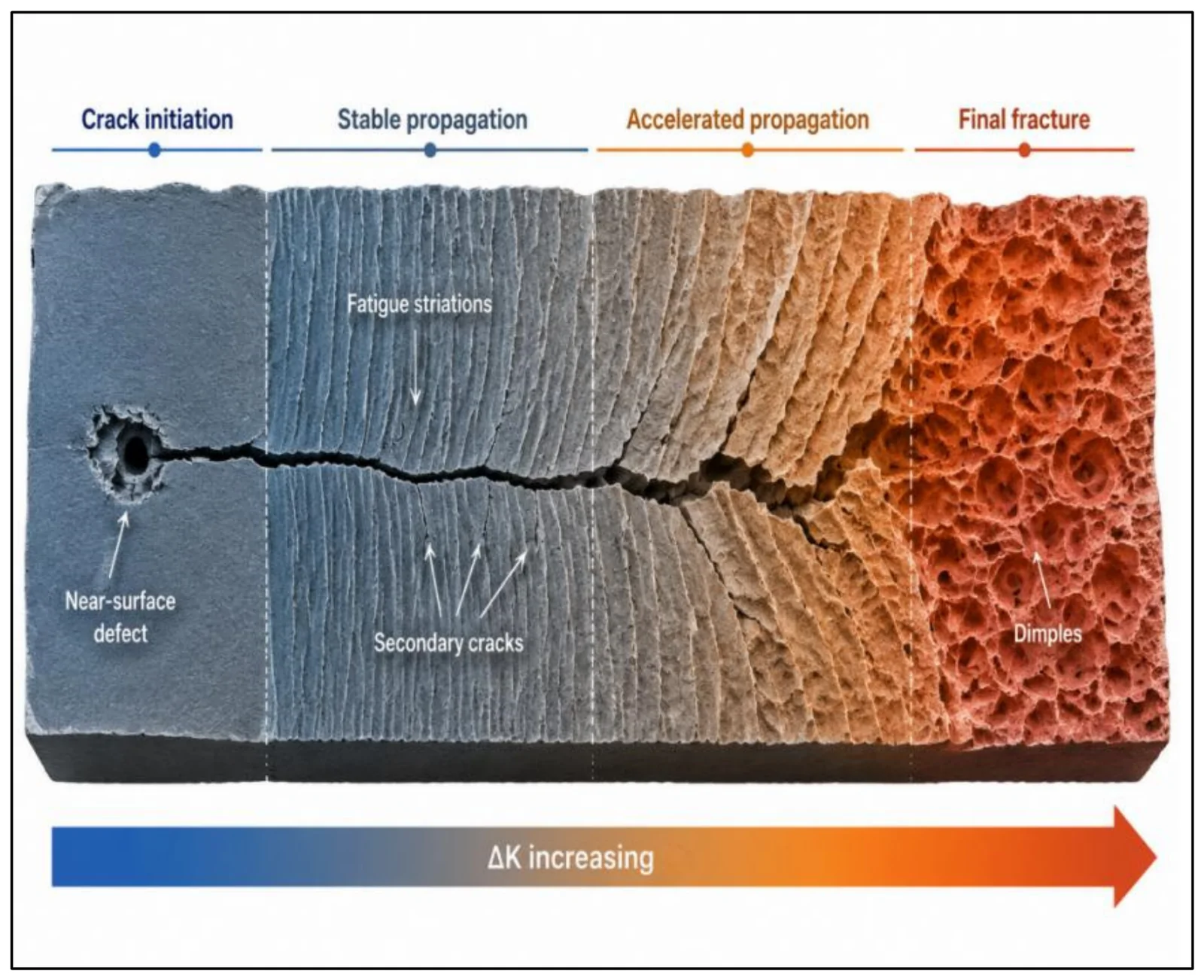
Schematic illustration of the three stages of fatigue fracture and the corresponding fracture-surface zones. The schematic was initially drafted with the assistance of AI and subsequently redrawn and finalized by the authors.

As shown in Figure 11, the fatigue fracture surface can be divided into crack-initiation, stable-propagation, and final-fracture regions. The initiation region is usually associated with low ΔK values and may contain facets, quasi-cleavage features, and early fatigue striations, whereas the stable-propagation region is characterized by fatigue striations and secondary cracks. During cyclic tensile loading, local strain concentration near fatigue striations can promote the formation of secondary cracks that are often aligned with or parallel to the striation direction [348]. Secondary cracks can partially shield the main crack by dissipating energy and redistributing local stresses around the crack tip [349]. Tear ridges can appear in both crack-initiation and propagation regions, indicating localized plastic deformation and ligament tearing. The formation of rough crack-propagation features may be associated with high stress-intensity factors, which indicate severe stress concentration at the fatigue-crack tip and can promote crack-path deflection under normal loading [350]. The fatigue crack-propagation zone can be further divided into an accelerated crack-growth region and a final fracture region at high ΔK, where pitted or dimple-like features are often observed, indicating a ductile overload-fracture mode [351]. The fatigue-crack growth rate increases with load ratio and, under the reported conditions, reaches its maximum when the loading direction is parallel to the deposition direction (θ = 0 degree), indicating pronounced anisotropy. This anisotropy is associated with orientation-dependent grain refinement induced by interlayer cooling and with directional residual-stress distributions; in some directions, finer grains and residual compressive stress can retard crack propagation [173,352,353].

**Figure 12:**
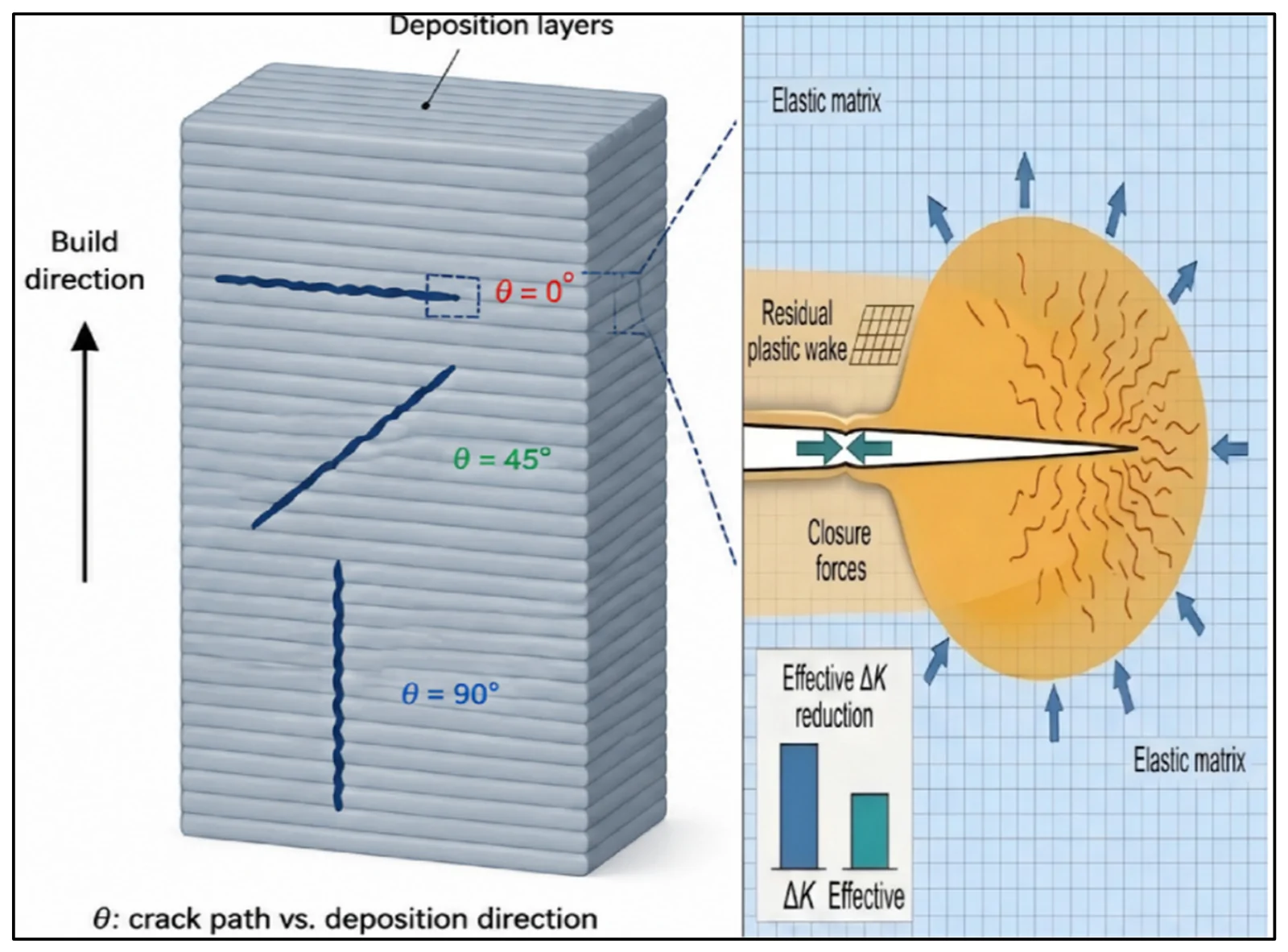
Mechanism map of fatigue-crack-growth anisotropy and plastic wake. Note: AI was used only for text-based conceptual suggestions; the figure was fully created by the authors.

High ductility in the diagonal direction (θ = 45°) can also inhibit fatigue-crack propagation. Ductility strongly affects fatigue-crack propagation because greater crack-tip plasticity can enhance plasticity-induced crack closure [351]. During cyclic loading, the local stress concentration at the crack tip causes permanent plastic deformation and generates large plastic strains. Part of this plastic strain can be retained during unloading, forming a plastic wake behind the crack tip, as shown in Figure 12. Ajay et al. [354] reported that, in the diagonal direction of structural components, the combination of high ductility (up to 92%) and high yield strength generated pronounced plastic wakes and strong crack-closure effects, thereby enhancing resistance to fatigue-crack propagation. Changes in strength and elastic properties therefore have a strong influence on the fatigue performance of manufactured parts [9,355]. Strength, particularly yield strength and ultimate tensile strength, is crucial for resisting fatigue-crack initiation and sets the upper limit for fatigue-stress levels [356]. Blinn et al. [357] optimized the design and manufacturing process by aligning the principal load-bearing direction diagonally within the deposited part. This diagonal alignment improved tensile and yield strength and consequently enhanced fatigue strength and service life compared with vertically or horizontally built specimens, indicating the importance of orientation design in WAAM components. The elastic modulus reflects the overall stiffness and cyclic stress response of the material [358]. A reduced elastic modulus may indicate high residual porosity within the specimen, although texture and phase constitution may also contribute [352]. These pores reduce strength and act as fatigue-crack initiation sites, thereby strongly affecting fatigue life. For AM austenitic stainless steels, fatigue-fracture morphology should therefore be interpreted by correlating fracture-surface features with defect type, defect location, surface condition, residual stress, and microstructural anisotropy.

#### 4.3.2. Assessment of Fatigue Fracture

**Figure 13:**
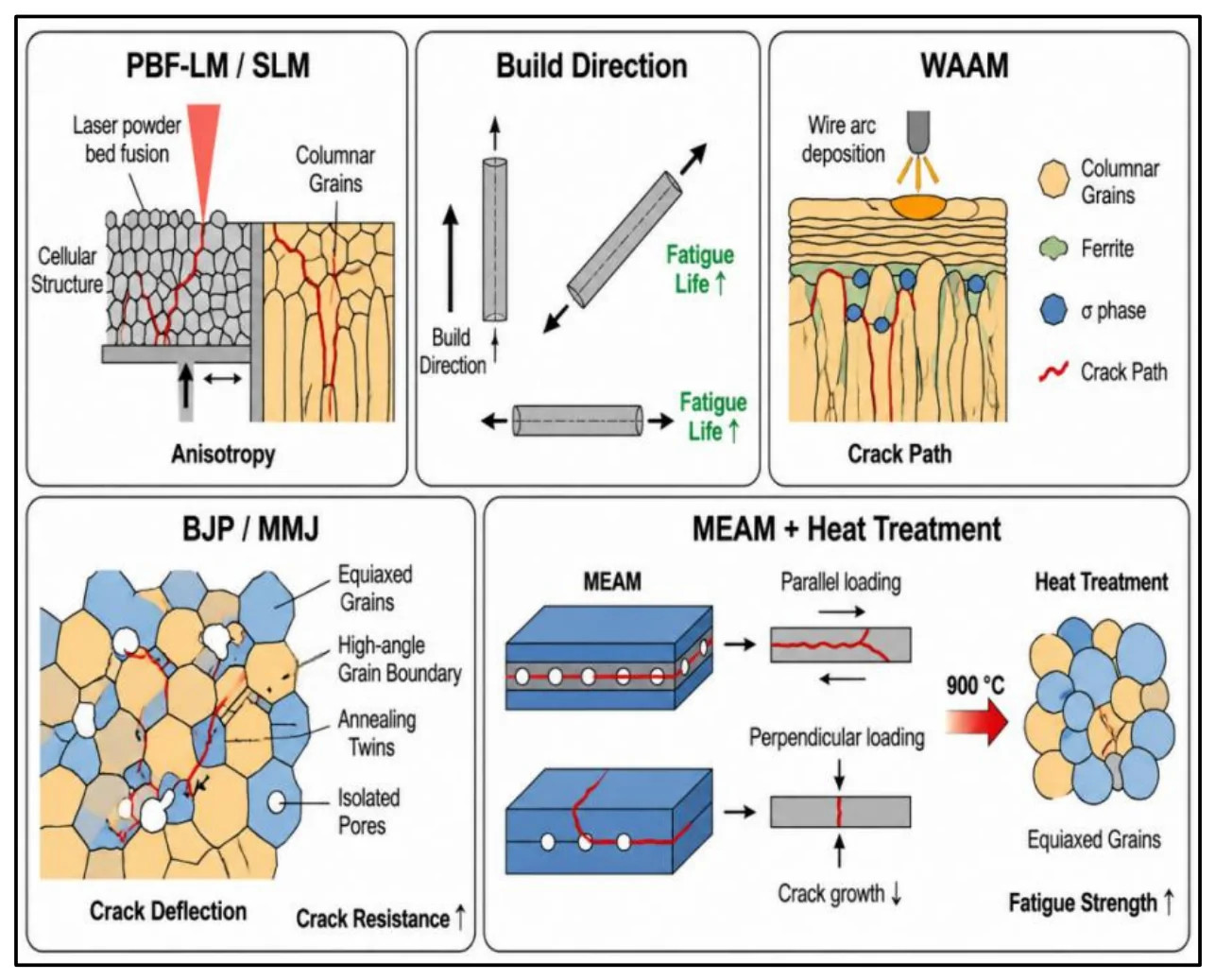
Schematic illustration of the effects of AM process characteristics on microstructure, defects, crack-propagation behavior, and fatigue performance of austenitic stainless steels. Note: AI was used only for text-based conceptual suggestions; the figure was fully created by the authors.

Fatigue assessment is crucial for predicting damage accumulation, crack initiation, and crack propagation in additively manufactured austenitic stainless steels under cyclic loading. The evolution of these fatigue-damage processes ultimately determines fatigue resistance and service life [359,360]. Traditional models, such as the stress–life method based on S–N curves [361] and the strain–life method based on ε–N curves [362], rely heavily on extensive experimental data and empirical calibration. However, their predictive capability is limited for AM materials, particularly under complex loading paths, anisotropic microstructures, and defect-sensitive fatigue conditions [363,364]. Recent advances in computational modeling, data science, and in situ monitoring have promoted the development of multi-scale and multi-physics fatigue assessment methods, as shown in Figure 13. These approaches can improve the efficiency and accuracy of fatigue-performance evaluation for AM metallic materials through multi-scale characterization and multi-physics monitoring [347,365,366,367].

In terms of the interaction between surface morphology and defects, Parareda et al. [368] investigated the coupling between surface topography and defects using a stepwise loading program combined with digital image correlation. By monitoring the total strain range and its inelastic component in real time, they identified three stages: micro-damage initiation, micro-crack propagation, and macro-crack formation. This approach enabled rapid estimation of the fatigue limit based on the damage-evolution rate and cyclic inelastic strain. Bemani et al. [367] monitored changes in specimen stiffness and plastic-strain accumulation during a stepwise load-increase fatigue test. They defined the stress amplitude corresponding to a sudden stiffness drop as the fatigue limit, obtaining only a 2.4% deviation from the conventional step method and indicating the reliability of the approach. Zhang et al. [369] explored thermodynamic dissipation mechanisms by tracking the evolution of two-dimensional temperature fields in real time using infrared thermography. By applying thermodynamic principles, they quantified internal dissipation and linked it to microscopic plastic deformation, thereby improving the efficiency of fatigue assessment. Blinn et al. [357] combined the load-increase test (LIT) with the PhyBaLLIT life-calculation program. Using a limited number of specimens, they evaluated the effects of anisotropy and process differences on high-cycle fatigue performance and constructed S–N curves for different build directions. In cyclic constitutive modeling, Xiong et al. [358] developed a cyclic elasto-plastic constitutive model incorporating an anisotropic yield criterion, a strain-amplitude memory surface, and an improved kinematic hardening law. They characterized yield-surface evolution using a coefficient matrix related to plastic strain, enabling the simulation of hysteresis loops and plastic-strain accumulation in specimens with different orientations at elevated temperatures. The fatigue behavior of additively manufactured austenitic stainless steels is strongly influenced by defects, while the dominant defect type depends on loading mode, temperature, environmental medium, and surface condition. Under room-temperature high-cycle fatigue conditions, surface roughness, lack-of-fusion defects, and near-surface pores often serve as primary crack-initiation sources. Under thermomechanical fatigue conditions, inclusions, oxides along cellular boundaries, and local microstructural mismatch are more likely to induce void nucleation and crack coalescence. In corrosion-fatigue environments, local passive-film instability interacts with defect-size effects; large process-induced defects may dominate crack initiation over smaller secondary inclusions. Therefore, fatigue-performance evaluation should consider the specific loading condition, environment, surface state, and defect-size distribution. Overall, fatigue assessment of AM austenitic stainless steels is shifting from empirical life-curve fitting toward defect-sensitive, orientation-dependent, and multi-physics approaches that combine accelerated testing, in situ monitoring, and constitutive modeling.

#### 4.3.3. Influence of Process Characteristics

Different AM processes significantly influence the microstructure, defect characteristics, and residual-stress state of austenitic stainless steels, as shown in Figure 13. These variations arise from process-specific energy input, deposition path, thermal history, and post-processing condition, leading to differences in crack initiation, crack propagation, and fatigue life. For instance, L-PBF forms fine cellular substructures because of its high cooling rate. Such microstructures may exhibit strong anisotropy and promote fatigue-crack propagation along columnar grain boundaries or cellular walls, especially when defects and residual tensile stresses are not effectively controlled [352,370]. Build orientation also affects fatigue performance; horizontal or oblique specimens have been reported to exhibit a 100–196% increase in fatigue life compared with vertical specimens, owing to more favorable grain orientation and defect distribution under specific testing conditions [346]. Subsequent heat treatment, such as treatment at 900 °C, can weaken cellular substructures, relieve residual stresses, and promote equiaxed grain formation, thereby improving fatigue strength; in some cases, a nearly twofold increase has been reported [370].

In material extrusion additive manufacturing (MEAM), fatigue performance strongly depends on the relationship between the deposition direction and loading direction. When the loading direction is perpendicular to the material-deposition direction, fatigue performance can improve. Conversely, when the loading direction is parallel to the deposition direction, the directional pore network formed during printing can provide connected crack-propagation paths, thereby reducing fatigue resistance [352]. Arc-based additive manufacturing, characterized by high heat input and slow cooling, tends to produce coarse columnar austenitic grains and consequently strong mechanical anisotropy. Under multiaxial fatigue loading, cracks can propagate rapidly along columnar grain boundaries when the maximum shear-stress direction aligns with the grain-growth direction, thereby significantly reducing fatigue life [362]. Although WAAM can produce an austenite–ferrite duplex structure, thermal cycling may partially dissolve δ-ferrite and promote precipitation of the brittle σ phase. This degrades microstructural uniformity and accelerates fatigue-crack growth [371]. Despite the relatively high porosity of binder jetting (BJP) and metal material jetting (MMJ) processes, with BJP porosity reaching 3.7–5.6%, the equiaxed grains, high-angle grain boundaries, and annealing twins formed during sintering can effectively hinder crack propagation. Accordingly, the fatigue strength of BJP materials can be comparable to that of austenitic stainless steels produced by laser powder bed fusion (L-PBF) under the reported testing conditions [352,372]. Through powder refinement and process optimization—for example, by using submicron powders in MMJ—an ultrafine equiaxed grain structure with an average grain size of about 2.43 μm can be obtained. This can further increase the fatigue limit to about 241 MPa, indicating enhanced fatigue resistance [372]. These results indicate that fatigue performance cannot be assessed solely by porosity level; grain morphology, grain-boundary character, twin density, and crack-path tortuosity must also be considered. Overall, process optimization for fatigue resistance should not be based solely on reducing defect density; it should also coordinate build orientation, residual-stress control, surface finishing, microstructural homogenization, and post-processing strategy according to the intended loading path and service environment.

#### 4.3.4. Influence of Microstructure

Microstructural anisotropy in AM austenitic stainless steels reflects process-induced inhomogeneity and can modify crack-propagation paths, local stress states, and stress-intensity factors at the crack tip [266]. Li et al. [362] demonstrated that, under torsional loading, the alignment of columnar grains across layers with the maximum shear-stress direction promotes rapid crack propagation along grain boundaries, thereby reducing fatigue life. Retained δ-ferrite at grain boundaries may improve fatigue-crack resistance when it is appropriately distributed, because it can deflect crack paths, trap plastic deformation, and provide local bridging. However, owing to crystallographic, chemical, and deformation incompatibility with the austenitic matrix, the body-centered cubic δ-ferrite phase can impede dislocation motion and promote strain localization under cyclic loading [373].

As reported by WANG Q et al. [373], dislocations can accumulate around interdendritic δ-ferrite, storing strain energy at the γ/δ interface and making these regions susceptible to secondary fatigue microcrack initiation [371,374]. Fatigue strength often decreases with increasing ferrite volume fraction when ferrite forms continuous networks or promotes interfacial strain incompatibility. When δ-ferrite transforms into brittle σ phase or into electrochemically active regions under corrosive conditions, crack propagation can be accelerated, leading to degraded toughness and fatigue performance [351]. Li et al. [375] found that WAAM 302 stainless steel exhibits poorer crack-initiation resistance under low-cycle fatigue because multiple initiation sites, such as slip bands, austenite grain boundaries, and ferrite regions, can form. However, its coarse columnar grain structure can create a more tortuous crack-propagation path, thereby improving resistance to crack growth. In addition to the coexistence of δ-ferrite and austenite, metastable austenite may undergo strain-induced martensitic transformation, typically γ → α′ or γ → ε → α′, during cyclic plastic deformation at room temperature [371].

Martensitic transformation can substantially modify the cyclic mechanical response of AM austenitic stainless steels, depending on austenite stability, strain amplitude, and fatigue regime. In low-cycle fatigue, strain-induced martensitic transformation can delay crack initiation through the TRIP effect and work hardening, thereby improving low-cycle fatigue resistance [376]. In high-cycle fatigue, excessive or localized martensite may act as a stress-concentration site and brittle phase, promoting crack initiation and rapid propagation and thereby reducing fatigue performance [377]. Additionally, highly oriented nanotwinned layers can absorb deformation energy and inhibit fatigue-crack propagation [378]. As an alternative deformation mechanism, mechanical twinning can reduce the tendency for martensitic transformation, redistribute dislocation slip through grain reorientation, and improve fracture toughness [379]. Therefore, the effect of microstructure on fatigue should be evaluated by considering not only grain morphology and texture but also phase stability, ferrite morphology, interface compatibility, twinning behavior, and transformation-induced plasticity.

#### 4.3.5. Influence of Temperature

Temperature significantly influences the fatigue-fracture behavior of additively manufactured austenitic stainless steels, with mechanisms varying across temperature ranges and loading conditions. From room temperature to intermediate temperatures, approximately 25–550 °C, AM 316L stainless steel can exhibit fatigue performance comparable or even superior to that of wrought counterparts. This behavior is partly attributed to strengthening from cellular dislocation structures and high-density dislocation networks. However, above approximately 600 °C, fatigue life generally decreases and the dominant damage mechanisms shift toward thermally assisted softening, oxidation, and creep–fatigue interaction.

**Figure 14:**
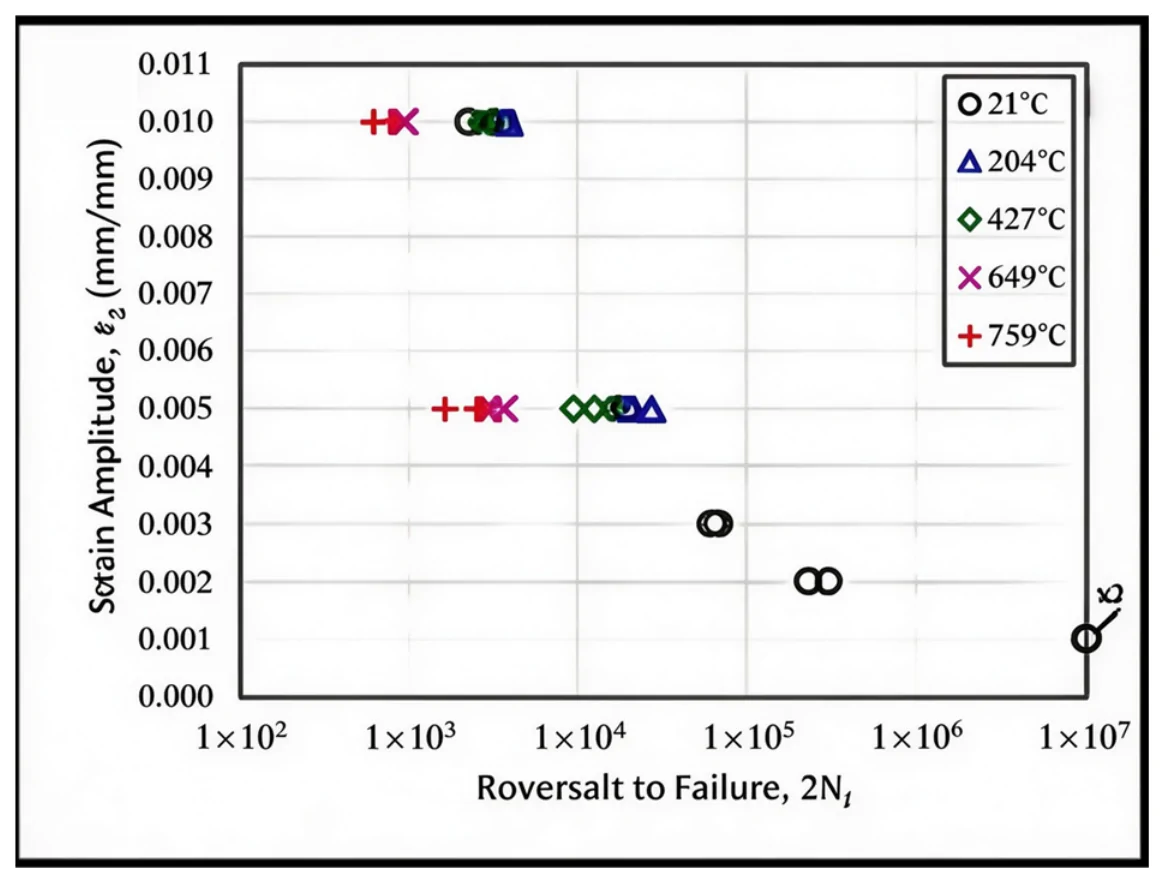
Fatigue behavior under different temperature conditions: strain–life fatigue data of LP-DED and forged 316L stainless steel [380]. Reprinted from [380], Lee S et al., Additively manufactured 316L stainless steel via laser powder directed energy deposition (LP-DED): Mechanical properties at cryogenic and elevated temperatures, *International Journal of Fatigue*, published by Elsevier, 2024 (CC BY 4.0).

As shown in Figure 14, Lee et al. [380] found that, at 760 °C, the absence of stable persistent slip bands alters the crack-initiation mechanism, resulting in a shorter fatigue life than that observed in the intermediate-temperature range. Under in-phase thermomechanical fatigue loading, grain boundary creep-cavity nucleation and growth become dominant damage modes, leading to intergranular fracture and a marked reduction in fatigue life. Conversely, under out-of-phase thermomechanical fatigue loading, oxidation-assisted transgranular fatigue-crack growth tends to prevail [381,382]. This distinction indicates that thermal–mechanical phase relationship, rather than temperature alone, can determine the dominant fatigue-damage mode. Additionally, Pal et al. [383] observed that the low-cycle fatigue life of laser directed energy deposition (L-DED) 316L at 500 °C is notably lower than that at room temperature, with fatigue cracks often initiating from internal lack-of-fusion defects. Xiong et al. [358] reported that, at 393 °C, horizontally built specimens exhibited higher stress amplitudes and lower ratcheting strains because of their higher yield strength, indicating pronounced anisotropic cyclic plastic behavior. Temperature strongly affects fatigue-damage mechanisms and fatigue life in AM austenitic stainless steels by controlling dislocation mobility, microstructural stability, oxidation behavior, and defect sensitivity. Therefore, temperature-dependent fatigue performance should be discussed by distinguishing strengthening-dominated behavior at low-to-intermediate temperatures from oxidation-, recovery-, and creep-assisted damage at elevated temperatures, while identifying the microscopic damage mechanisms specific to each temperature range.

#### 4.3.6. Influence of Inclusions and Reinforcing Particles

**Figure 15:**
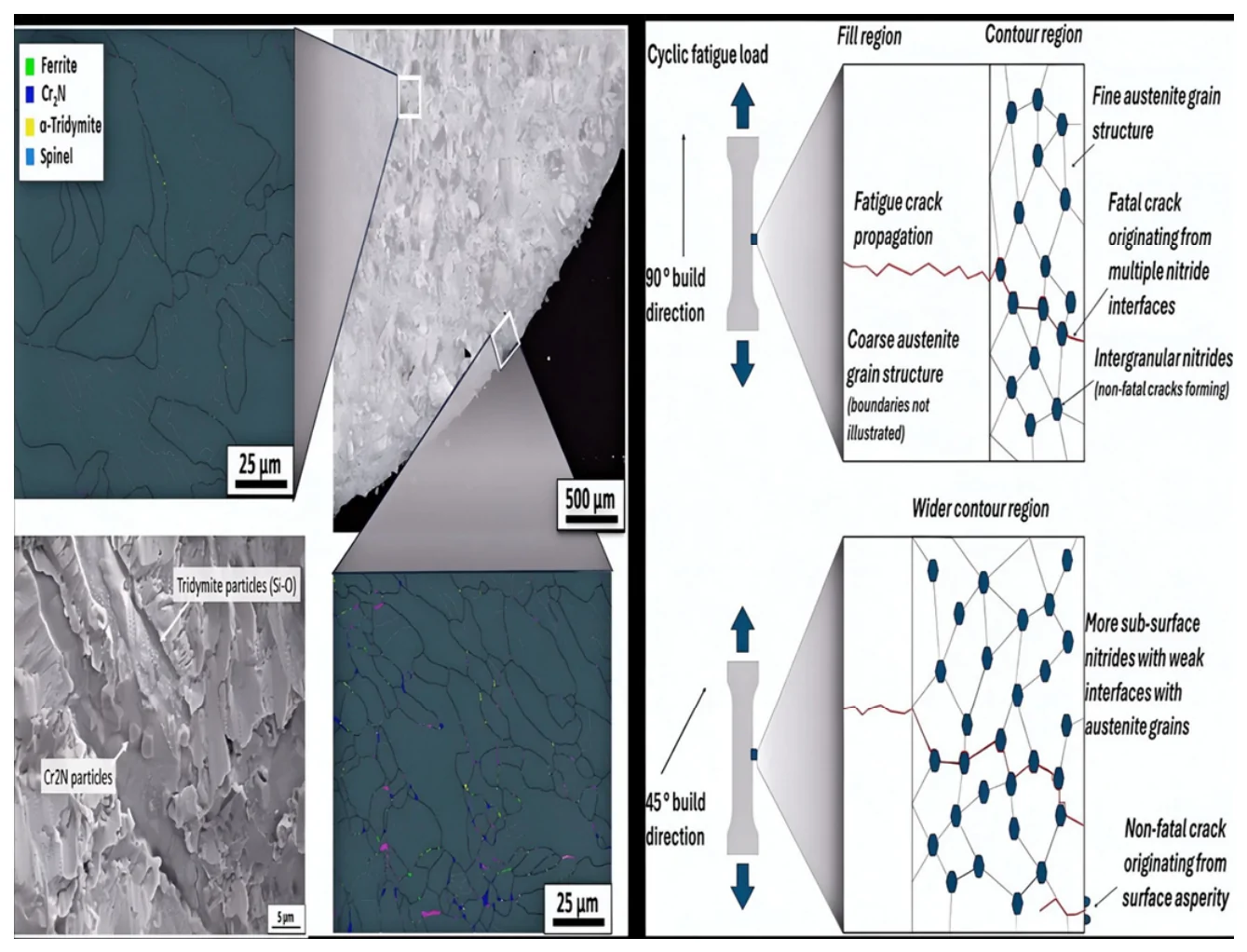
Secondary phases and fatigue-crack initiation: distribution of secondary phases along austenite grain boundaries; and schematic illustration of the fatigue-crack initiation mechanism inferred from fracture morphology [384]. Reprinted from [384], Wietecha-Reiman I J et al., Contributions of sub-surface intergranular phases on fatigue crack initiation in additively manufactured austenitic stainless steel, *Journal of Materials Science*, published by Springer Nature, 2025 (CC BY 4.0).

In additively manufactured austenitic stainless steels, inclusions and oxides are common microdefects that can strongly affect fatigue-crack initiation and propagation. As shown in Figure 15, these particles are typically distributed within the matrix, at grain boundaries, and along cellular boundaries, with sizes ranging from nanometers to the submicrometer scale. They can act as potential fatigue-crack nucleation sites and accelerate damage accumulation. During cyclic loading, inclusions and oxides generate local stress concentration because of elastic–plastic mismatch with the matrix, thereby promoting early microcrack initiation.

For instance, Babinský et al. [382] reported that spherical Mn–Si oxides are randomly distributed in the microstructure of AM 316L stainless steel. These oxides, together with interfaces such as dislocation-cell walls, can become nucleation sites for creep voids under thermomechanical fatigue, promoting internal crack networks under in-phase loading and reducing fatigue life. Similarly, Lou et al. [385] observed Si-rich and Mn-rich oxides distributed within grains and at grain boundaries. During fatigue, microcracks tend to initiate at inclusion/matrix interfaces, accelerating crack propagation and reducing fracture toughness. In addition, segregation of these second-phase particles at grain or cellular boundaries can increase the tendency for brittle or intergranular fracture. Reiman et al. [384] demonstrated that submicron Cr_2_N particles and nanoscale α-quartz oxides are mainly enriched at austenite grain boundaries in the subsurface contour region, forming a grain-boundary decoration structure. This promotes the initiation and propagation of intergranular brittle fracture, especially at low build angles. Kong et al. [71] further highlighted that Mn-, Si-, Ti-, and Al-rich oxides enriched at cellular boundaries, together with associated brittle phases, can become microcrack nucleation sites because of strain incompatibility under tensile stress. This results in a higher fatigue-crack growth rate in L-PBF 316L stainless steel compared with forged counterparts. However, in certain environments, the influence of inclusions may be overshadowed by other factors. Stern et al. [266] indicated that, although nanoscale silicate inclusions in AM AISI 316L are distributed at grain boundaries and cell walls, cracks under sodium chloride corrosion-fatigue conditions are more likely to initiate from surface or near-surface process defects, such as lack-of-fusion defects. Under corrosion-fatigue conditions, nitrogen alloying can indirectly mitigate the negative influence of inclusions on fatigue life by improving repassivation ability and pitting resistance. This suggests that, in corrosive environments, the interaction between defect size and environmental sensitivity may dominate fatigue behavior, making the role of fine inclusions relatively secondary. Therefore, inclusions should be evaluated not only by their size and chemistry but also by their spatial location, interfacial bonding, fracture susceptibility, and interaction with the service environment.

#### 4.3.7. Influence of Post-Processing

**Figure 16:**
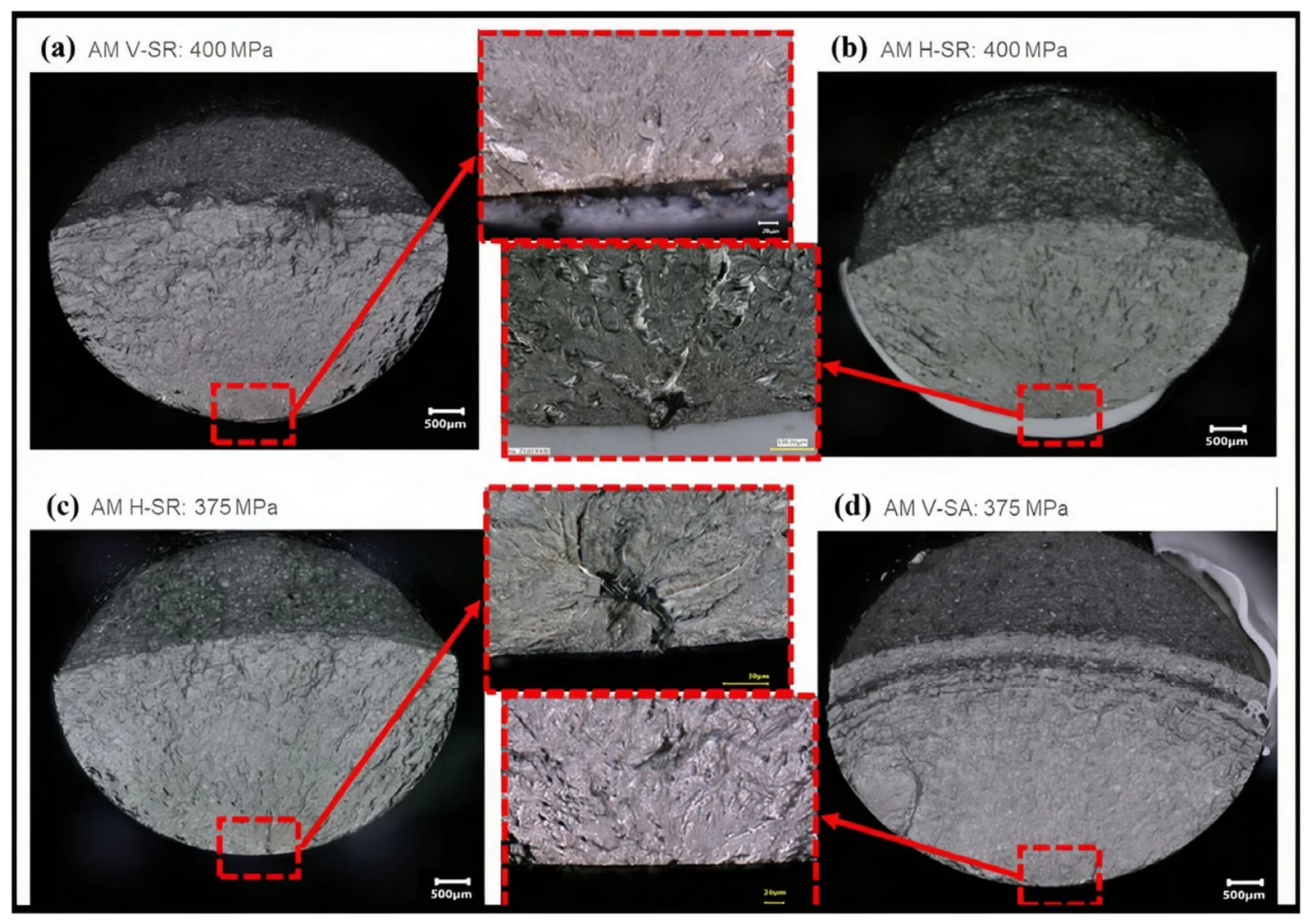
Fatigue fracture surfaces of additively manufactured 316L stainless steel under force-controlled fatigue tests: (**a**) AM V-SR at σa = 400 MPa; (**b**) AM H-SR at σa = 400 MPa; (**c**) AM H-SR at σa = 375 MPa; and (**d**) AM V-SA at σa = 375 MPa [393]. Reprinted from [393], Kluczyński J et al., Crack Growth Behavior of Additively Manufactured 316L Steel—Influence of Build Orientation and Heat Treatment, *Materials*, published by MDPI, 2020 (CC BY 4.0).

Post-processing can play a crucial role in enhancing the fatigue performance of additively manufactured austenitic stainless steels. Under cyclic loading, fatigue failures in AM components often originate from the initiation and propagation of surface or near-surface cracks [347]. Techniques such as polishing [385,386], shot peening [387], and vacuum laser treatment [328] can reduce surface roughness, mitigate near-surface defects such as cracks and pores, and modify the near-surface microstructure through mechanisms such as martensitic transformation, grain refinement, and formation of high-density low-angle boundaries. Some of these treatments, especially mechanical surface treatments, can introduce gradient residual compressive stresses in the subsurface, delaying crack initiation and extending fatigue life [388,389]. Heat treatment can improve the fatigue strength of AM austenitic stainless steels when it relieves detrimental residual stresses, homogenizes microstructure, or introduces beneficial boundary structures without excessive softening or brittle-phase precipitation. Wang et al. [390] found that grain coarsening induced by high-temperature heat treatment at 1100 °C can promote crack-tip blunting in the low-ΔK region. As shown in Figure 16, heat treatment can also modify the fatigue fracture morphology of additively manufactured austenitic stainless steels. The reduction in yield strength can enhance plasticity-induced crack closure, while increased high-angle grain boundaries and twin boundaries can further impede crack propagation. Yang et al. [218] noted that solution treatment at 1100 °C promotes grain homogenization and introduces numerous annealing twins, thereby decreasing the crack-growth rate. Gordon et al. [391] explained that cracks typically originate from slip bands, austenite–ferrite interfaces, or ferrite regions. Stress-relief heat treatment may remove beneficial compressive residual stresses that retard crack propagation, thereby increasing the fatigue-crack-growth rate under certain conditions. However, internal defects often serve as the primary crack-initiation source, making it difficult for post-processing heat treatment alone to effectively improve fatigue resistance in such cases [326,375,392]. Therefore, the effect of heat treatment should be further distinguished according to temperature range because stress relaxation, carbide precipitation, recovery, recrystallization, and grain-boundary evolution may contribute differently to fatigue resistance.

The influence of low- and medium-temperature heat treatments on fatigue performance varies because different temperature ranges induce distinct microstructural evolution pathways. Low-temperature treatments below 500 °C, such as 470 °C for 5 h, primarily promote dislocation rearrangement and partial residual-stress relaxation. However, the retained dislocation-cell structure limits further strengthening or microstructural homogenization [394]. In contrast, medium-temperature treatments between 500 and 700 °C, such as 650 °C for 1 h, fall within the carbide-precipitation-sensitive range. Carbides such as Cr_23_C_6_ may precipitate along grain boundaries, causing local Cr depletion and accelerating crack propagation under fatigue loading, especially in corrosive environments [390,395]. Treatments at sub-recrystallization temperatures, approximately 800–950 °C, such as 900 °C for 1 h, can partially relieve residual stresses but do not achieve full recrystallization. This may increase local microstructural inhomogeneity and does not effectively reduce susceptibility to crack initiation [356,370]. Therefore, fatigue-oriented post-processing should be selected according to the dominant crack-initiation mechanism: surface finishing is preferred for roughness-controlled fatigue, mechanical surface treatments are effective for surface-crack suppression through compressive residual stress, and heat treatment is suitable for residual-stress relief and microstructural homogenization when strength loss and brittle-phase formation are controlled.

Overall, fatigue performance is governed by multiple factors, including surface condition, internal defects, residual stress, and microstructure, rather than by a single microstructural factor. Under specific conditions, features such as fine grains, high dislocation density, and tailored phase distribution can improve strength, corrosion resistance, wear resistance, or fatigue performance. However, these benefits may be negated, or even become failure risks, when accompanied by severe microstructural inhomogeneity, defect clustering, or inappropriate heat treatment. Thus, developing a cross-scale process–structure–defect–performance correlation model is crucial for precise performance control.

## 5. Summary, Challenges, and Outlook

### 5.1. Summary

This review summarizes recent progress in defect evolution, microstructure control, post-processing strategies, composition design, and service performance of additively manufactured austenitic stainless steels. Owing to rapid melting–solidification, non-equilibrium thermal history, and repeated thermal cycling, AM austenitic stainless steels exhibit defect populations and multiscale microstructural features that differ markedly from those of wrought or cast counterparts. Defects such as pores, lack-of-fusion, and cracks coexist with microstructural features including dislocation-cell structures, residual δ-ferrite, and grain-boundary characteristics, among others. These features are strongly coupled and collectively determine corrosion resistance, wear resistance, fatigue behavior, and environmentally assisted failure, although their individual effects depend on volume fraction, morphology, distribution, and service conditions. Recent advances in solidification-mode regulation, crystallographic-orientation control, grain-boundary engineering, composition tailoring, and post-processing optimization have expanded the potential of AM austenitic stainless steels for high-performance applications. Overall, their service performance is governed by the synergistic evolution of defects and microstructures under process-dependent thermal histories, rather than by any single factor. Establishing the intrinsic “process–defect–microstructure–performance” relationship is therefore essential for developing service-reliable and designable AM austenitic stainless steels.

### 5.2. Challenges

Despite rapid progress, several key challenges remain. First, most studies on pores, cracks, segregation, cellular structures, phase boundaries, and residual stresses are still largely qualitative, while multiscale quantitative models linking process parameters, thermal history, defect characteristics, microstructural heterogeneity, and performance remain insufficient. Second, the damage-evolution mechanisms of AM-specific microstructures and defects under complex service environments are not yet fully understood, particularly under multi-physics coupling conditions such as corrosion–fatigue, hydrogen–stress coupling, and wear–oxidation interactions. Moreover, post-processing and alloy design strategies remain strongly dependent on the initial microstructural state, processing route, and target service condition. Universal design criteria and unified evaluation frameworks for balancing strength, ductility, corrosion resistance, wear resistance, and fatigue resistance are still lacking.

### 5.3. Outlook

Future research should focus on process regulation, defect control, microstructure design, and performance-oriented manufacturing. Fundamental gaps remain in understanding damage initiation and evolution under realistic service conditions—particularly corrosion–fatigue, hydrogen–stress interactions, and wear–oxidation coupling, and the long-term stability of non-equilibrium phases—for hydrogen-energy, marine, nuclear and aerospace applications. Closing these gaps requires integrating in situ characterization, multi-physics simulation and data-driven methods into predictive models spanning the whole process chain. For example, in LPBF 316L, laser power and scanning speed set the volumetric energy density and melt-pool thermal history, which control lack-of-fusion and keyhole porosity; as shown in Section 2.1 and Section 4.3, these pores act as preferential fatigue-crack initiation sites. This chain can be modelled by linking process parameters to melt-pool history, pore population and morphology, and then to the fatigue limit, enabling fatigue-relevant process-window design. In WAAM 316L, heat input and interlayer temperature govern δ-ferrite fraction and connectivity, which (Section 3.1 and Section 4.1) determine solidification-cracking susceptibility, pitting and hydrogen-assisted cracking; a coupled model would map heat input to δ-ferrite state and then to corrosion and hydrogen-embrittlement resistance. For post-processing, HIP and recrystallization annealing (Section 3.4) close pores, dissolve δ-ferrite and coarsen grains, jointly shifting fatigue-crack growth and corrosion behavior; such a framework should predict these microstructural changes and their net effect so that post-processing routes can be tailored to service conditions. These examples illustrate that building such models requires combining thermal–fluid melt-pool simulation, CALPHAD phase prediction, defect-population statistics and machine-learning property mapping, so that the process–defect–microstructure–performance relationship becomes predictive rather than empirical. Translating this into practice further demands coordinated control of defects, grain morphology, phase constitution, grain-boundary character, residual stress and surface integrity, together with unified evaluation frameworks, reliable process windows, non-destructive testing standards and certification criteria. AM austenitic stainless steels are thus expected to evolve from merely formable toward serviceable, predictable and designable.

## Figures and Tables

**Table 1:** Chemical compositions of austenitic stainless steel grades commonly used in additive manufacturing (wt.%).

Grade	C	N	Mn	Si	Cr	Ni	Mo	P	S	Fe	Other	PREN
S304	0.08	0.1	2	1	18–20	8–10.5		0.045	0.03	Bal		19
S304L	0.03	0.1	2	1	18–20	8–12		0.045	0.03	Bal		19
S316	0.08	0.1	2	1	16–18	11–14	2–3	0.045	0.03	Bal		25
S316L	0.035	0.1	2	1	16–18	10–14	2–3	0.045	0.03	Bal		25
S321	0.08	0.1	2	0.75	17–19	9–12				Bal	Ti: 5 × (C + N) − 0.7	18
S347	0.08		2	0.75	17–19	9–13		0.04	0.3	Bal	Nb: 10 × C − 1.0	18
S308L	0.03		2	1	19–21	10–12		0.045	0.03	Bal		24
S201	0.15	0.25	5.5–7.5	1	16–18	3.5–5.5				Bal		19
S310	0.08		2	1.5	24–26	19–22				Bal		25
PREN = 1 × wt.% Cr + 3.3 × wt.% Mo + 16 × wt.% N [29]												

**Table 2:** Commonly used additive manufacturing methods for austenitic stainless steels: typical microstructures and defects.

Grade	Typical Process and Heat-Source Characteristics	Typical Microstructure and Defects	Main Consequences for Properties	References
S304/S304L	WAAM, L-DED; high heat input, low cooling rate, repeated thermal cycling	Columnar/equiaxed dendrites, residual δ-ferrite, Si-rich oxide inclusions; L-DED or solution treatment tends to equiaxed recrystallization with annealing twins; water quenching after solution treatment suppresses carbide precipitation, whereas slow cooling or sensitization may precipitate Cr-rich carbides at grain boundaries. Defects: gas porosity, lack-of-fusion, deformation/collapse due to heat accumulation, residual stress	Moderate strength, good ductility; anisotropy from columnar grains; δ-ferrite and Si oxides affect corrosion resistance; pores and residual stress affect fatigue performance	[30,31,32,33]
S316/S316L	LPBF, WAAM, L-DED, BJP; LPBF: very high cooling rate, steep thermal gradient; WAAM/L-DED: low cooling rate, high heat input; BJP: sintering, low cooling rate	LPBF: cellular substructures, columnar grains, strong <001>/<110> texture, residual δ-ferrite. WAAM/L-DED: columnar grains, {100} texture, δ-ferrite. BJP: fine equiaxed (or bimodal) grain structure after sintering; residual porosity. HIP: recrystallized equiaxed grains, δ-ferrite dissolution, possible σ phase, oxide coarsening. Defects: gas porosity, keyhole pores, lack-of-fusion, residual porosity, oxide inclusions	LPBF: high strength/hardness but anisotropic, fatigue sensitive to defects; WAAM/L-DED: lower strength, better ductility, anisotropic; BJP: high porosity, lower fatigue resistance; HIP improves density but may cause grain growth or σ phase formation	[33,34,35,36,37]
S308/S308L	WAAM, DED; high heat input, low cooling rate, layer-by-layer thermal cycling	Columnar/equiaxed dendrites; microstructural gradient along build direction (finer equiaxed at bottom, coarser columnar at top); δ-ferrite increases toward top. Defects: deformation/collapse due to heat accumulation, gas porosity, MnS inclusions	Good ductility; anisotropy due to columnar grains and δ-ferrite gradient; corrosion and fatigue properties vary with location	[33,38,39,40]
S347	WAAM; high heat input, low cooling rate, repeated thermal cycling	Columnar + equiaxed dendrites; residual δ-ferrite varies along build direction; NbC precipitates; strong <001> texture and hardness gradient along build direction. Defects: gas porosity, lack-of-fusion, deformation/residual stress due to heat accumulation	Good high-temperature strength; anisotropy; hot corrosion/oxidation resistance affected by NbC and δ-ferrite	[41,42,43,44,45]
S321	LPBF, WAAM; LPBF: high cooling rate; WAAM: low cooling rate, high heat input	LPBF: cellular + columnar substructures; austenite with trace martensite; low residual δ-ferrite. WAAM: equiaxed, columnar or elongated dendrites; higher δ-ferrite fraction. Defects: gas porosity, lack-of-fusion, keyhole pores, microstructural anisotropy and columnar grains	LPBF: higher strength, lower ductility, anisotropic; WAAM: better ductility but lower strength; corrosion and fatigue affected by δ-ferrite and defects	[46,47,48]
S310	WAAM; high heat input, low cooling rate	Predominantly fully austenitic columnar grains; minor δ-ferrite may be present along dendrites or grain boundaries. Defects: gas porosity and microcracks	Good corrosion resistance but anisotropic; microcracks may reduce fatigue performance	[49,50]
S201	AM studies limited; data mainly from GTAW/PAW or laser welding; high heat input, low cooling rate	Columnar grains dominant; δ-ferrite distributed in dendritic or vermicular forms. Defects: not systematically documented; expected porosity, lack-of-fusion, and anisotropy inherited from WAAM/GTAW deposition	Corrosion resistance affected by δ-ferrite morphology and distribution; microstructure and properties under AM conditions still require further verification	[51,52,53]

**Table 3:** Effects of alloying elements on the corrosion behavior of additively manufactured austenitic stainless steels.

Element	The Impact on Additive Manufacturing Steel
Mo	Molybdenum affects passive-film thickness, stability, and repassivation behavior. It improves pitting corrosion resistance by stabilizing the protective passive film and promoting repassivation [267]. However, excessive Mo may promote carbide or intermetallic precipitation at grain boundaries, such as M_2_C-type carbides, thereby increasing the risk of intergranular corrosion [268].
Cr	Chromium promotes the formation of a dense Cr_2_O_3_-rich passive film, thereby improving corrosion resistance. It also increases ferrite stability and influences the γ/δ phase balance [269].
N	Nitrogen stabilizes austenite, reduces Cr segregation, and promotes the formation of a uniform Cr_2_O_3_-rich passive film. As an interstitial solid-solution element, nitrogen strengthens the matrix and can improve corrosion resistance by stabilizing austenite and enhancing repassivation [266]. Nitrogen-containing species, such as NH_4_^+^, can buffer local acidification and promote passive-film repair and stabilization [158]. However, excessive nitrogen, particularly under inappropriate processing conditions, may induce pore defects and promote chromium nitride formation, thereby accelerating localized corrosion [268].
Ni	Nickel stabilizes austenite, suppresses martensitic transformation, and improves phase stability, thereby reducing transformation-induced cracking susceptibility and indirectly supporting passive-film stability [65].
Mn	Manganese acts as an austenite stabilizer, but it can combine with sulfur to form MnS inclusions, which serve as preferential sites for pitting nucleation and reduce corrosion resistance [270].
O	Oxygen introduced during AM can form hard and brittle oxide inclusions, which disrupt passive-film continuity, act as crack-initiation sites, and reduce corrosion resistance [271].
S	Sulfur promotes MnS inclusion formation, providing preferential sites for pit nucleation and reducing both corrosion resistance and mechanical integrity [270].
H	Hydrogen ingress can alter passive-film chemistry by reducing the fraction of protective metal oxides, such as Cr_2_O_3_, and increasing hydroxide content, thereby decreasing film compactness and stability. It may also promote martensitic transformation or deformation twinning in metastable austenite, reduce toughness, and accelerate corrosion-crack propagation [272].

## Data Availability

No new data were created or analyzed in this study. Data sharing is not applicable to this article.
